# Application of Approved Cisplatin Derivatives in Combination Therapy against Different Cancer Diseases

**DOI:** 10.3390/molecules27082466

**Published:** 2022-04-11

**Authors:** Dobrina Tsvetkova, Stefka Ivanova

**Affiliations:** 1 Department of Pharmaceutical Chemistry, Faculty of Pharmacy, Medical University-Sofia, Dunav Str. 2, 1000 Sofia, Bulgaria; 2 Department of Pharmaceutical Chemistry and Pharmacognosy, Faculty of Pharmacy, Medical University-Pleven, Kliment Ohridski Str. 1, 5800 Pleven, Bulgaria; stefania_ivanova@yahoo.com

**Keywords:** metal complexes, Cisplatin derivatives, pharmacological activity, approved drugs, combinations, sinergism

## Abstract

The problems with anticancer therapy are resistance and toxicity. From 3000 Cisplatin derivatives tested as antitumor agents, most of them have been rejected, due to toxicity. The aim of current study is the comparison of therapeutic combinations of the currently applied in clinical practice: Cisplatin, Carboplatin, Oxaliplatin, Nedaplatin, Lobaplatin, Heptaplatin, and Satraplatin. The literature data show that the strategies for the development of platinum anticancer agents and bypassing of resistance to Cisplatin derivatives and their toxicity are: combination therapy, Pt IV prodrugs, the targeted nanocarriers. The very important strategy for the improvement of the antitumor effect against different cancers is synergistic combination of Cisplatin derivatives with: (1) anticancer agents—Fluorouracil, Gemcitabine, Cytarabine, Fludarabine, Pemetrexed, Ifosfamide, Irinotecan, Topotecan, Etoposide, Amrubicin, Doxorubicin, Epirubicin, Vinorelbine, Docetaxel, Paclitaxel, Nab-Paclitaxel; (2) modulators of resistant mechanisms; (3) signaling protein inhibitors—Erlotinib; Bortezomib; Everolimus; (4) and immunotherapeutic drugs—Atezolizumab, Avelumab, Bevacizumab, Cemiplimab, Cetuximab, Durvalumab, Erlotinib, Imatinib, Necitumumab, Nimotuzumab, Nivolumab, Onartuzumab, Panitumumab, Pembrolizumab, Rilotumumab, Trastuzumab, Tremelimumab, and Sintilimab. An important approach for overcoming the drug resistance and reduction of toxicity of Cisplatin derivatives is the application of nanocarriers (polymers and liposomes), which provide improved targeted delivery, increased intracellular penetration, selective accumulation in tumor tissue, and enhanced therapeutic efficacy. The advantages of combination therapy are maximum removal of tumor cells in different phases; prevention of resistance; inhibition of the adaptation of tumor cells and their mutations; and reduction of toxicity.

## 1. Introduction

Malignant cancers include a group of more than 100 different types of diseases, causing mortality worldwide. Carcinogenesis is a transformation of cells into tumors. This multi-stage process consists of initiation, promotion, malignant transformation of cells and progression [1]. During tumorogenesis, various genetic and epigenetic tumor-specific mutations occur, which define the biological features of tumor growth: uncontrolled cell proliferation, invasion of neighboring tissues, and metastasis of cells to distant tissues [2]. Cancer cells are characterized by high mitotic and proliferative activity, have a shorter cell cycle duration and a lower rate of cell death [3]. The main difference between normal and formed in carcinogenesis tumor cells is continuous tumor growth, which cannot be controlled [4]. Highly differentiated cancers have slower growth, metastasize relatively less frequently and later, and are less sensitive to cytostatics. Poorly differentiated cells proliferate rapidly, metastasize to distant organs and are sensitive to cytostatics. At the molecular level, tumors are considered to occur as a result of a DNA-mutations. The main reasons for the transformation of normal cells into malignant ones are: conversion of proto-oncogenes into oncogenes, inactivation of tumor suppressor genes (p53, RAS) and dysregulation of positive and negative signals for proliferation [5,6]. Cancer treatment includes surgical methods, chemotherapy, radiotherapy, targeted therapy and immunotherapy [7]. Most cancer treatments show significant side effects and their effectiveness is affected by various factors, including tumor and drug type, stage of the disease, resistance to the drugs, and the individual characteristics of patients [8].

Platinum complexes are among the most widely used anticancer chemotherapeutics. The electronic configuration determines the use of Pt II and Pt IV compounds as antitumor agents. Upon oxidation, irradiation with light or electron bombardment of Pt II complexes, intermediates are formed, in which the platinum is in the unstable oxidation state (+III) and is stabilized by complexation with suitable ligands. Compared to Pt II and Pt IV, Pt III compounds exhibit in vivo intermediate behavior, in terms of kinetic inertness and thermodynamic stability. Mixed-valence Pt III–Pt II complexes with a polymer structure have high anticancer activity, but the inability in obtaining a precise chain length makes the creation of a drug based on this difficult. Pt III monomer complexes [9,10] and stable octahedral Pt III hematoporphyrin compounds with cytotoxic activity are promising in Cisplatin-resistant tumor cell lines [11].

## 2. Cisplatin Combinations for Anticancer Therapy

**Figure 1:**
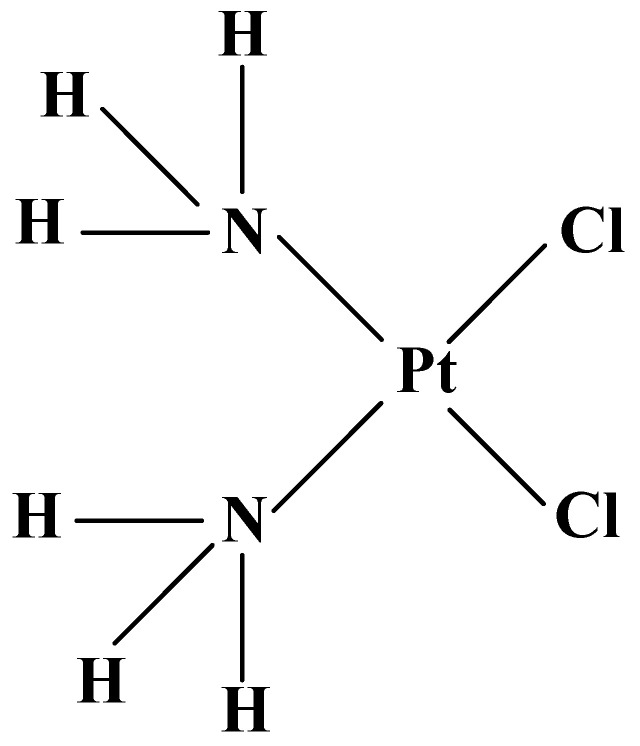
Chemical structure of Cisplatin.

The first platinum-containing coordination complex, applied for treatment of cancer, is Cisplatin (cis-dichlorodiamineplatinum II) (Figure 1).

Cisplatin has been approved from US Food and Drug Administration (FDA) in 1978 for therapy of testicular cancer [12,13]. Cisplatin is the most studied anticancer agent in clinical trials [14,15,16] and is applied as:

(1) a major component for the administration against ovarian carcinoma and testicular teratoma [17];
(2) first-line chemotherapy for head and neck [18], esophagus, gastric, colon, bladder, and cervix cancer [19];
(3) the second cytostatic towards advanced small-cell lung cancer (SCLC) and non-small-cell lung cancer (NSCLC), breast, pancreatic, liver, kidney, and prostate cancer [17], refractory non-Hodgkin’s lymphoma, sarcoma, neuroblastoma, malignant brain multiform glioblastoma, peritoneal and pleural mesothelioma, metastatic melanoma, leukemia [19];
(4) sensitization of tumor cells to radiation therapy for carcinomas of the head, neck, esophagus, lung, and gastric cancer [20].

In combination cancer treatment regimens, greater effectiveness is a result of the synergism of the different molecular mechanisms of drugs. In the therapy of first-line advanced NSCLC, the advantage of schemes is due to the inhibition of DNA-synthetic pathways, involved in the reduction of platinum-DNA adducts. Cisplatin is the most commonly used platinum-containing component in various treatment schedules, due to its synergistic effects with the following drugs: Capecitabine, Docetaxel, Epirubicin, Etoposide, Everolimus, Fluorouracil, Gemcitabine, Irinotecan, Nab-Paclitaxel, Paclitaxel, Vinorelbine, as well as with immunotherapeutic drugs such as Atezolizumab, Cemiplimab, Cetuximab, Durvalumab, Necitumumab, Rilotumumab, Trastuzumab, and with radiation therapy (Table 1).

The dose-limiting side effects of compound [45] are: neurotoxicity (peripheral neuropathy), nephro- [46,47,48], oto- [49], and gastrointestinal toxicity, and myelosuppression. Ototoxicity is a result from Cisplatin-induced apoptosis of sensory cells [49].

## 3. Anticancer Therapy with Approved Cisplatin Derivatives in Combination Regimens

The discovery of the mechanism of action and the main structure–activity dependencies of platinum complexes create opportunities for rational synthesis of new Cisplatin analogs as potential antitumor drugs with reduced resistance and toxicity and a wider spectrum of anticancer activity. The approach to the synthesis of such compounds includes the development of new structures, based on knowledge of the biochemical mechanism of Cisplatin action. The classic structural requirements for the manifestation of antitumor activity, derived on the basis of Cisplatin structure include:

(1) a flat-square Pt II complex
(2) cis-position of the two “leaving” ligands
(3) amino or imino ligands at the other two coordination site
(4) the presence of a NH-functional group in the platinum compounds, which is important for the stability of the formed adducts, due to additional binding via H-bonds

Prospects for the creation of new platinum complexes are various ligands—amines, alkylamines, purines, pyrimidines, hydantoines, carbonyl radicals—which produce compounds with selective cytotoxicity or inhibitory effect on resistant tumors. Modifications of leaving groups (labile, hydrolyzable or exchangeable ligands) in the main classes of platinum complexes, affect the cytotoxic activity, spectrum of action and the toxicological profile of platinum analogs. Suitable ligand systems have been selected, for obtaining effective accumulation in target tissues, reduced side effects, low nephrotoxicity. Strategies for the optimization of the effectivity of antitumor agents include [15]:

(5) Increasing of the therapeutic index towards Cisplatin-resistant ovarian, lung, breast, colon and prostate cancer
(6) Change of the degree of oxidation of the metal ion, for kinetic and thermodynamiccontrol over the binding of the metal complex to DNA bases and ensuring oralbioavailability—Satraplatin.

The development of platinum antitumor agents has been grown exponentially in recent decades. About 3000 derivatives have been synthesized and tested against cancer cells [35]. Only about 30 compounds have shown adequate pharmacological advantages over Cisplatin and have reached clinical trials, but most have been rejected, due to toxic effects [50,51].

Currently applied in clinical practice worldwide are: Cisplatin and its structural analog of II generation Carboplatin, derivative of III generation Oxaliplatin, Carboplatin derivative of II generation: Nedaplatin in Japan, Oxaliplatin derivatives—III generation: Lobaplatin in China and Heptaplatin in the North Korea. To date, no analog of Cisplatin has been developed that is completely superior to it, in terms of both therapeutic effect and spectrum of action [52].

**Figure 2:**
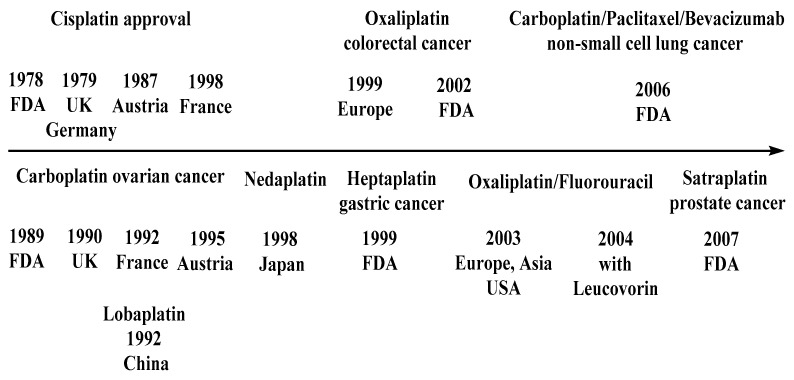
Regulations for Cisplatin and its derivatives in countries or regions.

The regulations for Cisplatin and its derivatives in countries or regions are presented on Figure 2.

### 3.1. Cisplatin Derivatives: Carboplatin and Oxaliplatin

#### 3.1.1. Carboplatin

**Figure 3:**
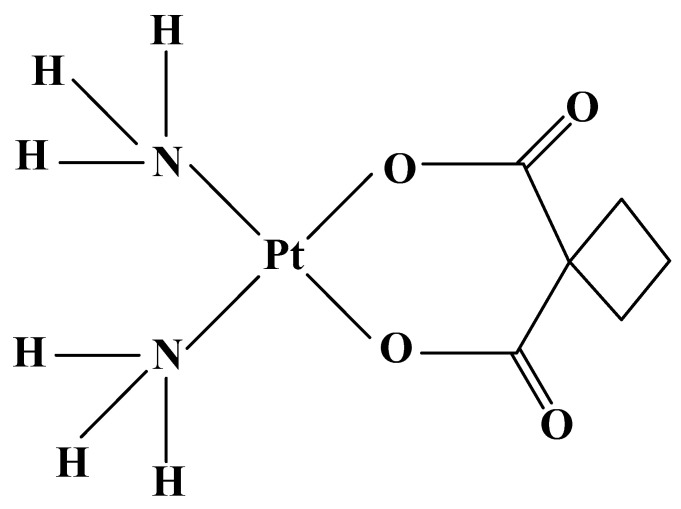
Chemical structure of Carboplatin.

Carboplatin (Paraplatin, JM 8) (Figure 3) is the most successful platinum complex of the second generation with clinical application. In 1989, the drug was approved by the FDA for use in advanced ovarian cancer and has been recommended for treatment of ovarian and testicular carcinomas only in cases, where there are abnormalities in renal function [53]. The compound possess significant activity in sensitizing tumor cells to radiation therapy for brain, head, neck, lung, breast, esophagus, bladder, cervical and salivary gland cancer [54], retinoblastoma, neuroblastoma, and nephroblastoma [14].

For the improvement of the antitumor effect on different types of cancer, combinations of Carboplatin with different drugsare applied: Amrubicin, Capecitabine, Docetaxel, Etoposide, Fludarabine, Fluorouracil, Gemcitabine, Irinotecan, Nab-Paclitaxel, Paclitaxel, Pemetrexed, Topotecan, Vinorelbine and immunotherapeutics Atezolizumab, Avelumab, Bevacizumab, Cetuximab, Durvalumab, Imatinib, Nivolumab, Trastuzumab, and Tremelimumab [55] (Table 2).

Compared to Cisplatin, the side effects of Carboplatin—neuropathy, nephro-, oto- and gastrointestinal toxicity—are less pronounced and more easily overcome, due to the slower hydrolysis of leaving bidentate dicarboxylate ligands, compared to labile chloride ligands of Cisplatin. A limiting side effect of compound is myelosuppression, clinically manifested as severe thrombocytopenia, neutropenia and leukopenia, which requires monitoring of blood parameters or dose reduction [14]. Although the low nephrotoxicity of Carboplatin is an advantage, especially in patients with kidney disease, it is not an alternative to Cisplatin in resistant tumors, due to the presence of cross-resistance [16].

#### 3.1.2. Oxaliplatin

**Figure 4:**
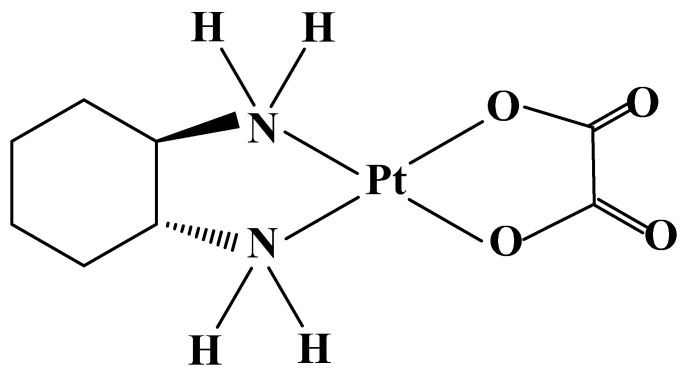
Chemical structure of Oxaliplatin.

Oxaliplatin (Figure 4) was synthesized in 1970 in Japan from Nagoya University [77]. The drug was approved by the FDA in the United States in 2002. The compound has a higher efficacy than Cisplatin, due to the additional inhibition of protein synthesis, which stops cell division. Oxaliplatin has been used in ovarian and breast cancers, non-Hodgkin’s lymphoma [78], glioblastoma, malignant melanoma, non-small-cell lung cancer [79], head and neck cancer [80], and neuroendocrine tumors [81].

Compared to Cisplatin and Carboplatin, the drug shows stronger activity, and an improved therapeutic index in colorectal tumors. The mechanism for this specificity is related to differences in absorption. The overexpressed in tumor cells transporting membrane proteins: human organic cationic transporters (OCTs) 1 and 2 (SLC22A1 and SLC22A2) are the major determinants of anticancer activity and contribute to antitumor specificity, and significantly increase the accumulation and cytotoxicity in cell lines of Oxaliplatin, but not of Cisplatin or Carboplatin. The compound in regimens with Fluorouracil has been approved in Europe, Asia, Latin America, and later in the United States (2003) for the treatment of metastatic colorectal cancer.

It has been reported that the antitumor efficacy of Oxaliplatin has been improved in schemes with the following drugs: Capecitabine, Cytarabine, Docetaxel, Epirubicin, Fludarabine, Fluorouracil, Irinotecan, Leucovorin, Trifluridine, and immunotherapeutic drugs Atezolizumab, Avelumab, Bevacizumab, Bortezomib, Durvalumab, Nivolumab, Onartuzumab, Panitumumab, Pembrolizumab, Rilotumumab, Sintilimab, Trastuzumab, and Tremelimumab (Table 3).

Oxaliplatin has less pronounced side effects, such as neutropenia, than Cisplatin, and the dose-limiting factors are significant neurotoxicity and tubular necrosis [103]. Neurotoxicity is: (1) initial transient peripheral sensory neuropathy, manifesting as paresthesias and dysesthesia in limbs and muscle contractions of the jaw; (2) long-term neuropathy, manifested by profound sensory loss, sensory ataxia, and functional impairment resulting from the ability to alter the potential of the outer surface membrane [104].

### 3.2. Carboplatin Derivatives: Nedaplatin 

**Figure 5:**
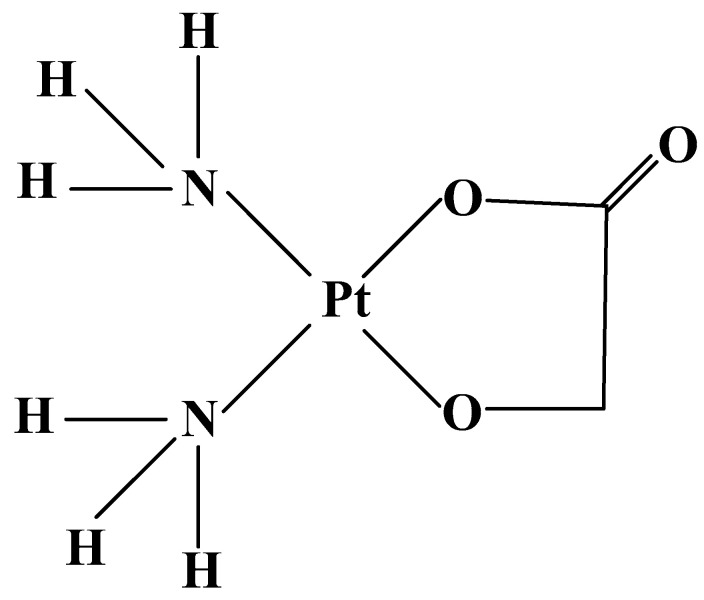
Chemical structure of Nedaplatin.

Nedaplatin (Figure 5) gives better results than Cisplatin in preclinical studies, and official indications in Japan are for the treatment of head, neck, esophagus [105], non-small-cell lung [106], cervical, testicular, and prostate cancer [105]. The drug does not demonstrate superiority over Cisplatin and Carboplatin, but shows a significantly better antitumor activity in the resected gynecologic carcinoma compared to Cisplatin. A synergistic effect towards cancer has been proven with drug combinations and with radiation therapy (Table 4) [107].

Nedaplatin has lower nephro- [136], neuro-, and gastrointestinal toxicity, and leukopenia. Dose-limiting toxicity is myelosuppression: thrombocytopenia, neutropenia, and anemia [137].

### 3.3. Oxaliplatin Derivatives—III Generation: Heptaplatin, Lobaplatin

#### 3.3.1. Heptaplatin

**Figure 6:**
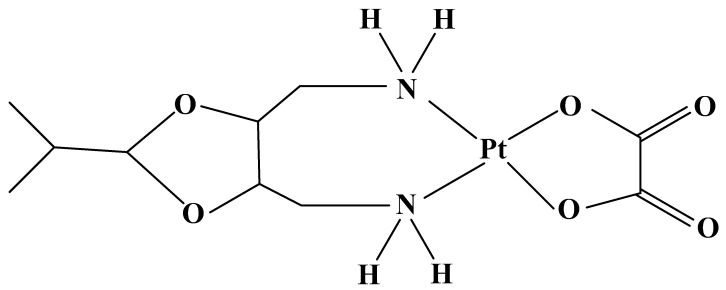
Chemical structure of Heptaplatin.

Heptaplatin (Figure 6) has been developed by the Sunkyong Industry Research Center (SK Chemicals, Kyungki-Do) in South Korea under the name SKI 2053 R [138]. The drug was entered in clinical trials in 1990 and was approved by the Korean Food and Drug Administration in 1999 under the name Sun Pla for the treatment of gastric cancer. The compound has greater antitumor activity in Cisplatin-resistant human gastric cell lines [139], and a weaker in vivo effect in small-cell lung cancer. Heptaplatin forms adducts, affecting DNA transcription and replication, and leading to cell death [140]. The drug has been studied in schedules with the following drugs:

(1) Fluorouracil in gastric cancer [141];
(2) Fluorouracil/Leucovorin in gastric cancer [142];
(3) Cucurbituril in colorectal tumor cells [143].

Heptaplatin has a lower effect than Cisplatin, but registration is based on its profile of lower nephrotoxicity and less frequent leukopenia, thrombocytopenia, neuro-, hepato-, and embryotoxicity [144].

#### 3.3.2. Lobaplatin

**Figure 7:**
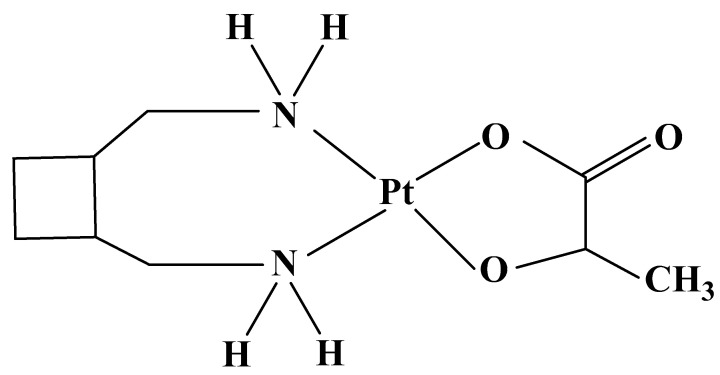
Chemical structure of Lobaplatin.

Lobaplatin (Figure 7) has been approved in China for the treatment of chronic myelocytic leukemia [145], hypopharyngeal carcinoma [146], esophageal squamous cell carcinoma [147], small-cell lung cancer [148].

The compound shows activity in various preclinical tumor models and has been observed to show potentialfor overcoming the resistance of some tumors to Cisplatin and Carboplatin. A second clinical phase has been completed in Australia, the EU, Brazil, and South Africa in studies of metastatic breast [149] andgastric cancer [150], and osteosarcoma [151]. By forming DNA adducts, the drug is active against Cisplatin- and Carboplatin-resistant tumors [152], and against a lung squamous cell line [153], cholangiocarcinoma cell line RBE [154], cervical cancer cell Line CaSki [155].

For improving the antitumor effect, Lobaplatin has been studied in regimens with the following drugs: Capecitabine, Docetaxel, Etoposide, Fluorouracil, Gemcitabine, Irinotecan, Leucovorin, Paclitaxel, Pemetrexed, and immunotherapeutics Bevacizumab, Temozolomide (Table 5).

Despite in vitro efficacy, in clinical trials in ovarian cancer with recurrence after first-line treatment with Cisplatin, the lower activity than Cisplatin has been reported. The approval of Lobaplatin is based mainly on reduced side effects: no nephro-, neuro-, and ototoxicity. The main side effects are thrombocytopenia, leucopenia, neutropenia, granulocytopenia, and anemia [167].

### 3.4. Derivatives of Cisplatin—Pt IV Complexes: III generation: Satraplatin

**Figure 8:**
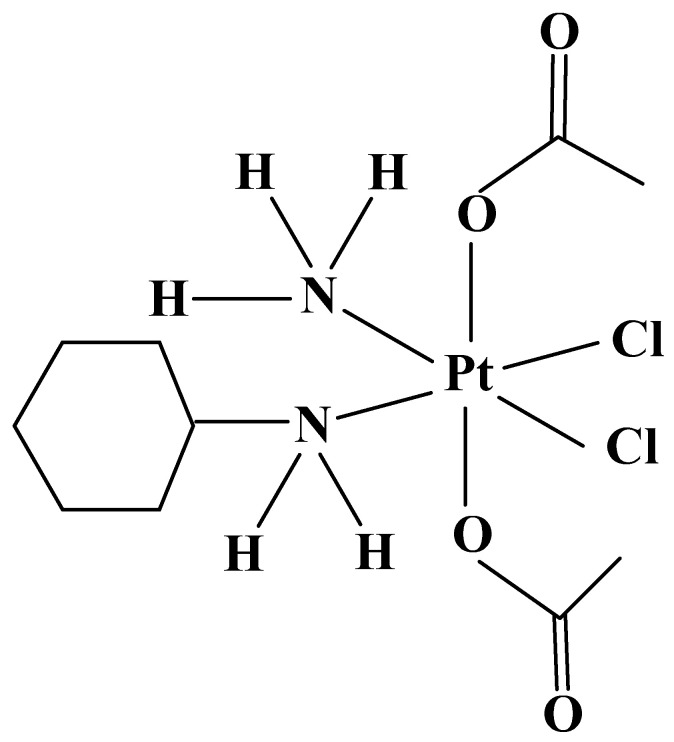
Chemical structure of Satraplatin.

Satraplatin (JM 216) (Figure 8) was developed by the Institute for Cancer Research in London and Johnson Matthey/AnorMed in 1992. The chemical structure differs with Cisplatin with two acetate groups and a cyclohexyl group, which increases lipophilicity and allows oral administration. Unlike the intravenous platinum analogs Cisplatin, Carboplatin, and Oxaliplatin, Satraplatin is the first orally active Pt IV chemotherapeutic drug [168] towards cell lines of cervical, prostate, and ovarian cancer. Satraplatin is not recognized by DNA repair proteins due to various adducts, DNA cannot be replicated and apoptosis occurs. Preclinical in vitro and in vivo studies have shown that the compound has good antitumor activity, comparable to intravenous Cisplatin or Carboplatin in breast, lung, prostate, ovarian [169], and brain cancer [170].

The drug has activity in cancer cells with Cisplatin resistance due to reduced platinum transport. Satraplatin synergistic combinations are with:

(1) Erlotinib for ovarian cancer [171];
(2) Bevacizumab [172], Docetaxel [173], Prednisone [174] for prostate cancer;
(3) Gemcitabine [175] for gastric, pancreatic, bladder, prostate cancer, hepatocellular carcinoma, and papillary renal carcinoma.

Studies with Satraplatin have revealed dose-limiting toxicities similar to those of Carboplatin with observed myelosuppression (thrombocytopenia and neutropenia), but no marked nephro-, neuro-, and ototoxicity have been reported [175].

## 4. Combination Therapy for Overcoming of Toxicity and Resistance to Cisplatin Derivatives

### 4.1. Toxicity of Cisplatin Derivatives

The toxicity is problem with chemotherapy with Cisplatin derivatives. Antitumor drugs have no selectivity for tumor cells, but also can kill normal cells with high proliferative activity: cells of the bone-marrow hematopoiesis, and in a gastrointestinal tract. Myelosuppression leads to a risk of infection. Despite its significant therapeutic efficacy, the clinical application of Cisplatin in effective high-dose courses is limited by the manifestation of severe dose-limiting side effects, as a result of an interaction with other biomolecules in the body, and the development of resistance, due to inefficient accumulation in tumor tissue.

In comparison with Cisplatin, the advantages for its derivatives are less pronounced side effects than Cisplatin, as follows [51,176]:

(1) neurotoxicity: Nedaplatin [105], Heptaplatin [144], Lobaplatin [152], Satraplatin [174];
(2) nephrotoxicity: Carboplatin [51], Oxaliplatin [103], Nedaplatin [105], Lobaplatin [152], Satraplatin [174];
(3) ototoxicity: Carboplatin [51], Lobaplatin [152], Satraplatin [174];
(4) gastrointestinal toxicity: Carboplatin [51], Nedaplatin [105];
(5) leukopenia, thrombocytopenia, hepato- [144], and embryotoxicity: Heptaplatin [177];
(6) neutropenia: Oxaliplatin [103].

Dose-limiting effects of approved Cisplatin analogs are presented in Table 6.

### 4.2. Resistance to Platinum Compounds

Anticancer chemotherapy with Cisplatin analogs is based on inhibition of the growth of the tumor cells, with DNA being the main target molecule, and is associated with growth inhibition of the tumor cells, due to suppression of DNA synthesis and repair as a result from modification of the three-dimensional structure of DNA, induced by metal adducts. The antitumor effect of Pt-containing drugs has been best studied for Cisplatin, which acts as a bifunctional alkylating agent on DNA. Cisplatin preferably binds to the N7 atom of the imidazole ring of the purine base guanine (G) of DNA and with N3 and 4-NH_2_ of cytosine and N1 and 6-NH_2_ adenine. Adducts cause disturbances in the structure of DNA: inhibit cellular processes of replication and transcription, cause prolonged G2 phase of cell cycle and lead to programmed cell death (apoptosis) [181]. Due toslower hydrolysis and the stability of carboxylate (Carboplatin) and oxalate (Oxaliplatin) ligands, these drugs bind to DNA more slowly [182].

Similar to Carboplatin, Nedaplatin is a precursor of Cisplatin, due to the formation of intermediate derivatives: monochloro-diamine platinum and dichloro-diamine platinum. The drug has 10 times greater solubility in water than Cisplatin, and is hydrolysed by double hydration to the same active metabolite as with Cisplatin: diaqudiamine-platinum. Nedaplatin binds to guanine in the DNA. In attempts of mismatch repair protein complex for repairing of the DNA by removing of the platinum cross-links, this complex forms single strand breaks and induces apoptosis after the repair attempt has failed. In combination with radiation treatment, the radiosensitizing effect of Nedaplatin is due to the formation of lethal double strand breaks [183]. For Heptaplatin, the platination processes is characterized by the binding to the guanine and adenine bases of DNA, forming the DNA-aducts. The binding of Heptaplatin hydrolytic products to guanine occurs more often in comparison with adenine, due to more favorable hydrogenbonds [184]. In the hydrolysis mechanism of Lobaplatin in aqueous medium under neutral conditions, the ring-opening reaction is found to be the rate limiting. The completely hydrolysed Lobaplatin reacts with the DNA purine base [185].

The problem with the chemotherapy is the resistance, which varies with different tumors: some, such as melanoma, adrenal, and lung tumors are difficult to respond to, and others become resistant during the treatment cycles, especially in cases of suboptimal doses. As a multifactorial process, the platinum drug resistance can occur through different mechanisms: decreased platinum drug uptake, improved drug efflux, intracellular detoxification by glutathione and metallothioneins, enhanced DNA repair, defective apoptosis, and modulation of signaling pathways. The mechanisms of tumor resistance to Cisplatin and its analogs can be summarized as caused by inhibition of platinum binding to DNA and mediated after DNA binding [186].

(A) Resistance, caused by inhibition of platinum binding to DNA is a result of the following:

(1) Insufficient influx and reduced cellular accumulation of Pt-compounds in cells, as a result of reduced transport across the cell membrane, and altered level of expression, localization or activity of multiple transporters, involved in the active transport of antitumor agents. The approach to overcoming of the resistance is the higher lipophilicity, which leads to greater accumulation of Pt-compounds in cells. Due to the interactions of Cisplatin with the copper transporters [187,188]: CTR1 [189,190], CTR2 [191], the overcoming of platinum resistance can be obtained using a copper-lowering agent [192];
(2) Increased efflux of the drug outside the cells.

Platinum drug resistance is dominated by improved extracellular transport through the participation of transporters: ATP7A, ATP7B, MDR1, and pumps for ATP-dependent glutathione S-conjugate: MRP1 (ABCC1), GS-X (MRP2, CMOAT, ABCC2), MRP3 (ABCC3), and MRP5 (ABCC5) [186].

(3) Enhanced system for the detoxification of Pt-compounds, by binding in the cytoplasm to complexes with reducing agents: glutathione, Methionine and metallothioneins, and prevention of the formation of DNA-Pt-adducts.

Dihydroxy-active metabolites have a high affinity for molecules with reducing properties, containing a thiol group: the tripeptide glutathione, Methionine, Cysteine, metallothioneins, and thioredoxin. The most common causes of resistance are high concentrations in the cytoplasm of glutathione and metallothioneins, which through sulfur-containing aminoacids Cysteine and Methionine react with activated aqua compounds to dysfunctional metabolites, reducing the possibility of interaction with DNA and leading to resistance.

(B) Resistance, mediated after DNA binding by the following:

(1) Increased capacity for recognition and removal from specific proteins of DNA-adducts and resynthesis of the damaged areas of DNA molecules;
(2) Modification of gene expression;
(3) Modulation of signaling pathways;
(4) Reduced apoptosis by inhibition of apoptotic genes or activation of anti-apoptotic genes [193];
(5) Autophagy [194], which determines resistance to Cisplatin in ovarian [195], and esophageal [196] tumors, and to Oxaliplatin in colorectal tumors [197].

Antitumor treatment may not be effective, due to low sensitivity of malignant cells to drugs or due to the adaptation of the cancer cells to the drug occurring during treatment. The adducts are recognized by cellular signal transduction proteins: p53 [198,199,200,201], p73, amino-terminal kinase JUN (JNK, MAPK8), which control cell growth and differentiation and participate in the repair of the damaged DNA. Cells respond to the structural deformation of DNA, caused by Pt-adducts, through protective and repair mechanisms, that regulate drug accumulation or detoxification, and lead to resistance to Pt-complexes. Cells with improved DNA repair activity are Cisplatin-resistant. The leaving group is important for the spectrum of platinum cytostatics and in the order: Cl-, oxalato-, cyclobutanedicarboxylato-, reduces cross-resistance in Cisplatin-resistant tumors [202].

Resistance in cancer cells might be either intrinsic to the tumors, as has been observed in pancreatic and colorectal carcinoma or might be developed during therapy, as has been for ovarian cancer [203]. p53-controlled apoptotic signaling plays an important role in the response of cancer types to chemotherapeutic drugs. The correlation between the p53 status and response of tumors to platinumanticancer drugs has been indicated. Better Cisplatin effect has been observed in patients with p53 wild-type tumors, and a poor response to the drug has been connected with the inactivation of p53 [204]. In contrast to most human cancers, p53 mutations are rare in testicular tumors, and this lack of p53 mutations is the reason for the better therapeutic response to therapy, due to the sensitivity of testicular tumors against Cisplatin [205]. In contrast, in 50% of colorectal cancers, mutations in the p53 gene have been identified [206] which lead to inactivation of p53, and due to this lack of function of p53 it has been observed that tumors with mutant p53 are more chemotherapy-resistant than those with wild-type p53 [207]. The development of Cisplatin resistance during the first-line treatment arises after prolonged use and causes cancer relapse, and results from alterations in DNA methylation, the inhibition of apoptotic genes, or the activation of antiapoptotic genes. Cisplatin resistance is mainly caused by the disruption of p53-mediated DNA damage. The resistance of pleural mesothelioma cells towards Cisplatin is promoted by the overexpression of inhibitors of apoptosis proteins [208]. The factors contributing to the formation of resistance to Carboplatin are enhanced efflux and reduced uptake of the drug, intracellular detoxification by glutathione, increased DNA repair, and defective apoptotic mechanisms [209].

The major way for the development of the resistance to Cisplatin and to its derivatives, is through a mammalian nucleotide excision repair pathway, which repairs the damaged DNA. A strategy for the reduction of the overall toxicity and resistance is the use of prodrugs, that can be activated locally and, being kinetically more inert than Pt II complexes, are expected to present fewer side effects [202].

The activation of Pt IV complexes is accomplished by reduction to Pt II, which is a necessary condition for achieving antitumor activity [14]. The reduction of Pt IV to Pt II is a necessary condition for achieving antitumor activity, as six-coordinate octahedral Pt IV compounds can minimize unwanted side reactions with biomolecules and are more kinetically inert and less reactive and more resistant to ligand substitution than four-coordinate Pt II [10]. In vitro studies have indicated that Pt IV complexes are characterized by higher efficacy than analogical Pt II complexes. From this class of platinum complexes is the first clinically administered oral drug: Satraplatin [210,211].

In comparison to other platinum anti-cancer drugs, due to the different adducts on the molecule (cyclohexamine), Satraplatin is not recognized by the DNA repair proteins, the DNA remains damaged, and DNA can not be replicated, resistance is solved. By binding to guanine residues, Satraplatin inhibits DNA replication and transcription, which leads to apoptosis [212].

### 4.3. Combination Therapy

Combination therapy is an important trend in the development of platinum anticancer agents and for bypassing of resistance to Cisplatin derivatives [55]. The need for the development of different cytostatics is a result ofthe data on the kinetics of tumor growth and from the comparative analysis of clinical outcomes in mono- and combination treatment. Today, the monotarget strategy is being replaced by a polytarget one, which achieves greater clinical efficacy in tumors. The administration of multiple anticancer regimens is a major strategy in oncology, and it is important to exploit the synergy between the drugs for a better outcome than monotherapy. The molecular mechanisms of drugs may differ; and their different schemes will combat cancer cells more effectively because they hit two or three different targets.

The development of drug schedules against malignant tumors is based on certaincriteria and principles:

(1) Simultaneous application of drugs with different mechanisms and targets to achieve a more pronounced cytotoxic effect on cancer cells and prevention of resistance, based on clinical observations that tumors resistant to one cytostatic may be sensitive to another type;
(2) Combinations for influencing different phases of the cell cycle, which enhances cytostatic activity by affecting many more cancer cells at different stages of development;
(3) Synergism of action of drugs in regimens on the same signaling pathway for the obtaining of a greater efficacy and more pronounced selectivity to the target, better outcome in more patients, eliminating to a greater extent the risk of rapid development of resistance, and achieving an impact on primary resistant tumor clones;
(4) Schemes of classical cytostatics with monoclonal antibodies for the improvement of the therapeutic response, higher frequency of remissions, and delay of progression.

The data reported in Table 1, Table 2, Table 3, Table 4 and Table 5 show that the more commonly applied therapeutic schedules of Cisplatin derivatives are with the following drugs: DNA alkylators, antimetabolites, topoisomerase inhibitors, steroids, tubuline inhibitors, DNA demethylating agent Decitabine). These are used for overcoming resistance in ovarian cancer, caused by hypermethylation of the MutL homolog-1 (MLH1) gene [213].

The different molecular mechanisms of anticancer drugs give the possibilities for obtaining synergistic effectsbetween drugs in a specific schedule, due to them simultaneously affecting DNA breaks or their associated processes (related proteins, specific genes) as targets for cancer therapy [214]. Combinations provide more effective treatment due to drugs affecting more than one different targets. Other anticancer mechanisms include the affecting of tumour cell membrane receptors and intracellular pathways (Table 7) [215].

The below tables summarize the most common regimens of Cisplatin and its derivatives with antimetabolites (Table 8), DNA topoisomerase inhibitors (Table 9), and tuboline inhibitors (Table 10).

Gimeracil is included in S-1 and is a reversible inhibitor of the liver enzyme dihydropyrimidine dehydrogenase and, due to this, it supresses the degradati on of Fluorouracil into inactive metabolites. Potassium oxonate preferentially localizes in the gut and inhibits the enzyme orotate phosphoribosyl-transferase [223].

The advantage of schemes of Cisplatin derivatives with antimetabolites is the synergistic effect, due to different molecular targets. Capecitabine is a prodrug that is selectively tumor-activated to its cytotoxic derivative Fluorouracil by thymidine phosphorylase, an enzyme found in higher concentrations in many tumors compared to normal tissues or plasma. Within normal and tumor cells, Fluorouracil is further metabolized to active metabolites: 5-fluoro-2′-deoxyuridine 5′-monophosphate and 5-fluorouridine triphosphate, which causes cell injury by the following mechanisms [244]: (1) 5-fluoro-2′-deoxyuridine 5′-monophosphate and the folate cofactor, N5-10-methylenetetrahydrofolate, form a covalently bound ternary complex, by binding to thymidylate synthase, which inhibites the formation of thymidylate from 2′-deaxyuridylate—thymidylate is a necessary precursor of thymidine triphosphate, and by its deficiency suppresses DNA replication and cell division [245]; (2) during the synthesis of RNA, nuclear transcriptional enzymes mistakenly incorporate 5-fluorouridine triphosphate in place of uridine triphosphate, and this metabolic error interferes with RNA processing and protein synthesis [244]. Pemetrexed is recommended in schedules with Carboplatin towards advanced non-small-cell lung cancer. By blocking the formation of precursor purine and pyrimidine nucleotides, the drug prevents the obtaining of DNA and RNA, which are required for the growth and survival of both normal and cancer cells. Pemetrexed disturbs enzymes, used in purine and pyrimidine synthesis: thymidylate synthase, dihydrofolate reductase, and glycinamide ribonucleotide formyltransferase [246].

**Table 9:** Combinations of Cisplatin and its derivatives with DNA topoisomerase inhibitors.

DNA Topoisomerase Inhibitors	Cisplatin and Derivatives
Cisplatin	
Benotecan	small-cell lung cancer [247]
Irinotecan	esophageal [216], and penile carcinoma [248]
Topotecan	squamous and non-squamous cervix carcinoma [209,222]
Etoposide	non-small-cell lung [249], small-cell lung [247], prostate [250], and germ-cell cancer [251]
Doxorubicin	endometrial cancer [252]
Epirubicin	prostate [250], and germ-cell cancer [253]
**Carboplatin**	
Irinotecan	small-cell lung cancer [65], esophageal carcinoma [216]
Topotecan	ovarian cancer [231], rhabdomyosarcoma [254]
Etoposide	small-cell lung [66], non-small-cell lung [249], and prostate cancer [250], esophageal carcinoma [216]
Amrubicin	small-cell lung cancer [65]
Doxorubicin	ovarian [231], and endometrial cancer [252]
Epirubicin	prostate cancer [250]
**Nedaplatin**	
Irinotecan	non-small-cell lung [111], small-cell lung [255], and testicular cancer [209], lung squamous cell [128], neuroendocrine lung [114], and endometrial carcinoma [133]
Topotecan	small-cell lung cancer [255]
Etoposide	lung carcinoma [114]
**Lobaplatin**	
Irinotecan	small-cell lung cancer [159]
Etoposide	small-cell lung cancer [158]

Etoposide forms a ternary complex with DNA and the topoisomerase II enzyme. Topoisomerase II normally forms a double-stranded break in one DNA double-strand, allows another to pass through, and relegates the broken strands. Etoposide’s binding prevents topoisomerase II from re-ligating the broken DNA strands. This results in a double-strand break in the DNA, errors in DNA synthesis, and promotes apoptosis of the cancer cell [256]. The inhibition of topoisomerase by the active metabolite SN-38 of camptothecin derivative Irinotecan leads to the suppression of both DNA replication and transcription [257]. Irinotecan stabilizes topoisomerase 1–DNA covalent complexes, leading to DNA strand breaks and cytotoxic damage in cells and, due to this mechanism, increases the effect of platinum drug/Etoposide [258].

**Table 10:** Combinations of Cisplatin derivatives with tuboline inhibitors.

Tubuline Inhibitors	Cisplatin and Derivatives
Cisplatin	
Docetaxel	non-small-cell lung [30], and breast cancer [259]
Paclitaxel	non-small-cell lung [249], bladder [221], cervical [222], and endometrial cancer [252], esophageal squamous cell carcinoma [31]
Vinorebine	non-squamous non-small-cell lung [24], small-cell lung [27], breast [260], and cervicar cancer [222], esophageal squamous cell carcinoma [216]
**Carboplatin**	
Docetaxel	non−small-cell lung [249], breast [261], ovarian [227], and prostate cancer [250]
Paclitaxel	non-small-cell lung [59,216], gastric [74], breast [262], bladder [221], ovarian [231], endometrial [252], and prostate cancer [263], urotelial [70], and esophageal squamous cell carcinoma [72,216],
Nab-Paclitaxel	breast cancer [264]
Cabazitaxel	prostate cancer [265]
Vinorebine	non-small-cell lung [64], and breast cancer [260]
**Oxaliplatin**	
Docetaxel	pancreatic adenocarcinoma [266]
Paclitaxel	non-small-cell lung [267], and ovarian cancer [268]
Vinorelbine	breast cancer [269]
**Nedaplatin**	
Docetaxel	non-small-cell lung [109], and breast cancer [260], squamous cellcarcinomas: oral [209], esophageal [116], and lung [126]
Paclitaxel	ovarian [131], cervical [134,135], and testicular cancer [209], esophageal squamous cell carcinoma [120]
Nab-Paclitaxel	non-small-cell lung cancer [112]
Vinorebine	non-small-cell lung [270], and breast cancer [260]
**Lobaplatin**	
Docetaxel	breast cancer [162]
Paclitaxel	gastric cancer [166], esophageal carcinoma [166]
Vinorelbine	breast cancer [260]
**Satraplatin**	
Docetaxel	prostate cancer [173]
Paclitaxel	non-small-cell lung cancer [271]

Tubuline contributes to mitosis, intracellular transport, and cell shape. The taxanes stabilize microtubules and, as a result, disturb the normal microtubule reorganization. The advantages of regimens of Cisplatin derivatives with tubulin inhibitors are in the application of drugs with different mechanisms of action. Docetaxel binds to the microtubules reversibly, leading to their stabilization. Paclitaxel binds to the beta-tubulin subunits of microtubules, and blocks spindle function by inhibition of microtubule dynamics and microtubule detachment from centrosomes. Paclitaxel stabilizes the microtubule polymer and protects it from disassembly. Chromosomes are unable to achieve a metaphase spindle configuration and this suppresses the progression of mitosis and cell division [272]. By binding to the specific sites on tubulin, the vinca alkaloids prevent polymerization of tubulin dimers, and disrupt the formation of microtubules. Vinca alkaloid Vinorelbine suppresses microtubule dynamics, resulting in mitotic block and apoptosis.

The advantages of double-drug schemes with Cisplatin derivatives is the increasing of effectivity and reduction of the dose of combined components. From the summarized data, it is obvious that other very-often-applied schedules are between Cisplatin derivatives and targeted drugs—signaling protein inhibitors and monoclonal antibodies. Specific targets for the development of new drugs are related to the specifics of tumor development, which determine their malignant phenotype: constant signals of continuous tumor growth, avoidance of antiproliferative control, tissue invasion and metastasis, and dysregulation of cellular metabolism. Targeted drugs affect post-genomic-specific targets—intracellular proteins, genes, or enzymes—that are involved in signaling continuous and uncontrolled cell growth, and correct cell dysregulation and genomic instability. Targeted inhibitors help for the achievement of better therapeutic results in malignant tumors that are difficult to treat with other therapies and are more suitable for maintenance therapy than cytostatics. Inhibitors of post-genomic signal transduction suppress proteins, that carry signals for the continuous and uncontrolled growth of cancer cells. They interact with growth protein receptors, containing tyrosine kinase, or with other molecules in the post-genomic signaling pathway. Their effect is in correction and normalization of cell function and contributes to stopping the progression of tumor growth. They are effective only in certain types of tumors with specific expression of the target molecule.

Tyrosine kinase inhibitors are a future direction for overcoming resistance. More than 700 tyrosine kinases, entering the structure of the kinase domain of growth factor receptors, have been identified in cells. These enzymes are important regulators of intracellular signaling pathways because they are involved in cell differentiation, intercellular communication, and phosphorylate tyrosine residues. The enhanced activity of tyrosine kinases and the mitogen-activated transduction protein kinase cascade is due to mutations, which are the main factors for uncontrolled tumor growth. Tyrosine kinase inhibitors interact with the ATP-binding region of the tyrosine kinase domain of growth factor receptors and interfere with the transmission of pathological signals. The proteasome is a complex of intracellular enzymes that break down tumor suppressor proteins in cancer cells, causing cell death, and this leads to the long-term vitality of tumor cells. Proteasome inhibitors stop this process by inhibiting the activity of the 20 S proteasome and tumor suppressor proteins, inducing apoptosis. The more-often-applied therapeutic regimens of Cisplatin derivatives are with the following targeted drugs:

(1) Erlotinib—monotarget tyrosine kinase inhibitor of BCR-ABL-protein receptors encoded by the Bcr-abl abnormal gene: Imatinib and of human epidermal growth factor type 1 EGFR (HER1);
(2) Bortezomib—proteasome inhibitor (apoptosis-inducing drugs);
(3) Everolimus—monotarget inhibitor of mTOR—target of rapamycin in mammals;
(4) Erlotinib—an epidermal growth factor receptor inhibitor and specifically binds reversibly to the adenosine triphosphate binding site of the epidermal growth factor receptor tyrosine kinase, which is highly expressed and occasionally mutated in various forms of cancer [257].

The boron atom in Bortezomib binds the catalytic site of the 26S proteasome with high affinity and specificity. In normal cells, the proteasome regulates protein expression and function by degradation of ubiquitylated proteins. Proteasome inhibition prevents the degradation of pro-apoptotic factors, thereby triggering programmed cell death in neoplastic cells [273].

Preclinical data suggest that platinum may be fully combined with the mammalian target of rapamycin inhibitors, such as Everolimus, which sensitizes cancer cells to Cisplatin by increasing Cisplatin-induced apoptosis.

Monoclonal antibodies are a human variants of large protein molecules of the immune system, that are designed to attack a specific target of a malignant cell. The first antitumor antibodies were directed against lymphoid antigens CD20 and CD52 (Alemtuzumab). Monoclonal antibodies are without much activity as monotherapy, but with an optimal effect in schemes with platinum drugs. The humanized monoclonal antibody Bevacizumab has not marked antitumor efficacy, but in 2006, was approved by the FDA in schedules for application with Carboplatin and Paclitaxel in recurrent or metastatic non-small-cell lung cancer. In advanced breast cancer, Trastuzumab in combination with Cisplatin, Carboplatin and Docetaxel has shown promising clinical activity.

A very important strategy for the improvement of the antitumor effect towards different cancer types is the application of the more effective synergistic regimens of Cisplatin derivatives with immunotherapeutic drugs such as: Atezolizumab, Avelumab, Bevacizumab, Cemiplimab, Cetuximab, Durvalumab, Erlotinib, Imatinib, Necitumumab, Nimotuzumab, Nivolumab, Panitumumab, Pembrolizumab, Rilotumumab, Trastuzumab, Tremelimumab, and Sintilimab [55]. The advantage of the application of Cisplatin derivatives in triples schemes or tetra-schedules is connected with more effective treatment, due to different mechanisms of drugs affecting different tumor targets.

Lung cancer is the most commonly diagnosed cancer (11.6%) and the leading cause of cancer death (18.4%) in both sexes, followed by breast (11.6%), prostate (7.1%), and colorectal cancer (6.1%) for incidence and colorectal (9.2%), stomach (8.2%), and liver cancer (8.2%) for mortality. Lung, breast, and colorectal cancers make upone third of worldwide cancer incidence and mortality and are the respective top three cancers in terms of incidence and within the top fivein terms of mortality: lung (first), colorectal (second), breast (fifth). Among males, lung cancer is the most frequent and the leading cause of death, followed by prostate and colorectal cancer (for incidence) and liver and stomach cancer (for mortality) [274].

Lung cancer is the leading cause of cancer incidence and mortality in men and women internationally and cases have increased two- to four-fold and have been found to be more aggressive in the HIV-infected population. Non-small-cell lung cancer (NSCLC) accounts for approximately 85%–90% of lung cancer diagnoses. Up to1990, squamous cell lung carcinoma was the most common subtype of lung cancers among men. Since 1990, due to genetic and hormonal factors, as well as oncogenic viruses, lung cancer incidences have risen and doubled in women; especially the higher are rates of adenocarcinoma relative to squamous and small-cell lung cancer. This is the reason why adenocarcinoma is the most common subtype of lung cancer in men and women today [275].

#### 4.3.1. Non-Small-Cell Lung Cancer (NSCLC)

For the first-line treatment of advanced NSCLC the most commonly applied double combinations leading to enhanced survival include platinum derivatives with one of the following drugs:

(1) alkylating agents: Ifosfamide, Mitomycin C;
(2) antimetabolites: Fluorouracil, Gemcitabine, Pemetrexed;
(3) inhibitors of topoisomerase I (Irinotecan), and topoisomerase II (Etoposide);
(4) microtubule stabilizers: Docetaxel, Paclitaxel, Nab-Paclitaxel;
(5) inhibitors of microtubule polymerization: Vinblastine, Vinorelbine [209].

Important double regimens for stage III of NSCLC are:

(1) Cisplatin with Gemcitabine [22], Etoposide [249] or Vinorelbine [24];
(2) Carboplatin with Fluorouracil [209], (S-1) [63], Gemcitabine [57], Etoposide [249], or Vinorelbine [64];
(3) Nedaplatin with Gemcitabine [110,127], Irinotecan [111], Docetaxel [109], or Nab-Paclitaxel [112].

The targeted therapy against stage III NSCLC depends on the type of genetic mutation found during diagnosis. The angiogenesis inhibitor Bevacizumab targets a protein vascular endothelial growth factor (VEGF), which helps new blood vessels grow [249]. In a phase 3 trial for advanced stage III NSCLC, the enhanced effectsof theaddition of the antibody Bevacizumab to Carboplatin/Paclitaxel have been reported [61]. Very promising is simultaneous administration of Nedaplatin/Paclitaxel with Docetaxel [83] or Sintilimab [113].

The following schemes have been approved for both stage III and IV of NSCLC:

(1) Cisplatin with Docetaxel [249], Paclitaxel [249], or Pemetrexed [216];
(2) Carboplatin with Docetaxel [276], Paclitaxel [59,216,277], or Pemetrexed [61];
(3) Cisplatin/Gemcitabine/Necitumumab [23].

The most important schedules for stage IV are: Cisplatin/Gemcitabine [234] and Carboplatin/Gemcitabine [57]. Different types of triple and tetra-drug combinations with addition of one or more drugs have been designed:

(1) Cisplatin/Ifosfamide with Etoposide or Vinblastine [278];
(2) Cisplatin/Mitomycin C with Ifosfamide or Vinblastine [276];
(3) Cisplatin/Gemcitabine with one of the following drugs: Paclitaxel [279]; Vinorelbine [279]; Erlotinib [280]; Bevacizumab [281]; Cetuximab [281]; Necitumumab [23];
(4) Cisplatin/Pemetrexed with Bevacizumab [282,283]; Ipilimumab; Ipilimumab/Nivolumab [284,285];
(5) Cisplatin/Docetaxel/Durvalumab [286];
(6) Cisplatin/Vinorelbine/Cetuximab [281];
(7) Carboplatin/Gemcitabine with Paclitaxel [287]; Avelumab [58];
(8) Carboplatin/Pemetrexed with Avelumab [58]; Ceritinib [288], Bevacizumab [283]; Pembrolizumab [289]; Ipilimumab; Ipilimumab/Nivolumab [284,285];
(9) Carboplatin/Paclitaxel with Bevacizumab [61], Cetuximab [277]; Bevacizumab/Cetuximab [277]; Nivolumab [62]; Ipilimumab; Ipilimumab/Nivolumab [284,285]; Veliparib [290];
(10) Carboplatin/Nab-Paclitaxel with Atezolizumab [60], or Pembrolizumab [216];
(11) Oxaliplatin/Cytarabin/Docetaxel [83].
(12) Nedaplatin/Paclitaxel/Sintilimab [113].

##### Non-Squamos Non-Small-Cell Lung Cancer (NS-NSCLC)

As first-line treatment in stage IV for metastatic non-squamous NSCLC, for obtaining an improved effect, is appropriate to adda monoclonal antibody to drug regimens, as follows:

(1) Sintilimab with Cisplatin/Pemetrexed [291];
(2) Camrelizumab [292] or Pembrolizumab [289] with Carboplatin/Pemetrexed;
(3) Bevacizumab [61,293]; Bevacizumab/Nivolumab [293]; or Motesanib [294] with Carboplatin/Paclitaxel;
(4) Atezolizumab [295] (blocks the PD-L1 protein on cancer cells) with Carboplatin/Nab-Paclitaxel [60];
(5) Tislelizumab/chemotherapy [296].

##### Squamous Non-Small-Cell Lung Cancer (S-NSCLC)

In patients with stage IV squamous NSCLC, the important role of additional drugs has been observed from the results of clinical trial. These drugs include Atezolizumab [297], Ipilimumab [298], Necitumumab [23], Pembrolizumab [299], Tislelizumab [300], Veliparib [301] for the greater anticancer activity of triple schemes in comparison with double schedules, as follows:

(1) Carboplatin/Paclitaxel with Ipilimumab [298] or Pembrolizumab [299];
(2) Carboplatin/Nab-Paclitaxel with Atezolizumab [297], or Pembrolizumab [299];
(3) Tislelizumab plus chemotherapy [300];
(4) Veliparib plus chemotherapy [301].

#### 4.3.2. Small-Cell Lung Cancer (SCLC)

Cisplatin/Vinorebine is applied both towards NSCLC [24], and SCLC [27]. For therapy of SCLC, the most often applied are:

(A) double combinations:

(1) Carboplatin with Vinorelbine [64], Irinotecan [65], Etoposide [66], or Amrubicin [65]
(2) Lobaplatin with Irinotecan [159], or Etoposide [158].

(B) triple and multiple regimens:

(1) Cisplatin/Etoposide with one of the following drugs: Ifosfamide, Irinotecan [25], Bevacizumab [302], Durvalumab [26,66], Durvalumab/Tremelimumab [66], Obatoclax mesylate [68];
(2) Carboplatin/Etoposide with one of the following drugs Atezolizumab [303]; Durvalumab [26,66]; Bevacizumab [302]; Durvalumab [66]; Durvalumab/Tremelimumab [66];
(3) Carboplatin/Paclitaxel/Ipilimumab [304].

The effectivity of double scheme Cisplatin/Etoposide is improved by simultaneous application of Irinotecan [25] or Durvalumab [26]. In the first-line treatment of the extensivestage of SCLC, the addition of Atezolizumab [303] to Carboplatin/Etoposide [26,67] increases the survival compared to monotherapy alone [67]. The effect of Carboplatin/Etoposide is improved from Durvalumab [26,66] and the efficacy of Carboplatin/Etoposide/Durvalumab is enhanced by combination with Tremelimumab [66].

For lung adenocarcinoma, the following importantschedules have been developed:

(1) platinum drug/Gemcitabine/Pembrolizumab [70];
(2) platinum drug/Docetaxel/Trastuzumab [71];
(3) Lobaplatin/Pemetrexed/Bevacizumab/Temozolomide [160].

#### 4.3.3. Esophageal Carcinoma

Gastric and esophageal cancer belongs to the most commonly diagnosed malignancies worldwide and are associated with a high disease-related mortality. Approximately 70% of cases of esophageal cancer occur in men [305]. Triple combination Fluorouracil/Leucovorin (Folinic acid): FOLFOX is applied for esophageal [82], gastroesophagealadenocarcinoma [86], and gastric cancer [94].

For the treatment of esophageal carcinoma, the administered double regimens are:

(1) Cisplatin with Capecitabine [216], Fluorouracil [216], S-1 [32], Irinotecan [216], Docetaxel [30], or Paclitaxel [31];
(2) Carboplatin with Fluorouracil [216], Irinotecan [216], or Paclitaxel [72];
(3) Oxaliplatin/Capecitabine [72];
(4) Nedaplatin with Fluorouracil [119], Docetaxel [116], or Paclitaxel [120];
(5) Lobaplatin with Fluorouracil [163], or Paclitaxel [166].

Antiproliferative activity against esophageal squamous cell carcinoma of the following double schemes has beenreported to be enhanced by simultaneous application of a third or fourth drug, as follows [216]:

(1) Cisplatin/Fluorouracil with Epirubicin; Doxorubicin; Mitomycin-C; Mitomycin-C/Cetuximab; Paclitaxel; Pembrolizumab [306]; Leucovorin/Etoposide [216];
(2) Cisplatin/Paclitaxel/Cetuximab [216];
(3) Carboplatin/Capecitabine with Paclitaxel or Docetaxel [216];
(4) Oxaliplatin/Capecitabine/Epirubicin (EOX) or EOX/Panitumumab [216];
(5) Oxaliplatin/Fluorouracil with Epirubicin (EOF); or Leucovorin (FOLFOX) [84];
(6) Nedaplatin/Nimotuzumab with Fluorouracil [121]; Gemcitabine ot Paclitaxel [121];
(7) Nedaplatin/Docetaxel [116] with Fluorouracil [117], or S-1 [118];
(8) Nedaplatin/Paclitaxel/Nimotuzumab [121].

#### 4.3.4. Gastroesophageal Adenocarcinoma

The most common triple schedule for the therapy of gastroesophageal adenocarcinoma isOxaliplatin/Fluorouracil/Leucovorin (FOLFOX) [86]. The increased effect has been obtained in simultaneousl application of Epirubicin with Cisplatin/Fluorouracil [34]. Docetaxel [33], or Epirubicin [34] with Cisplatin/Capecitabine. The survival of patients is enhanced in addition to Cisplatin/Capecitabine/Epirubicin of Rilotumumab [35], or Matuzumab [307]. Very promising is Carboplatin/Doxetacel/Capecitabine [73] and a triple combination in which Doxetacel [73], Epirubicin [73], or Sintilimab [85] enhance the effectivity of Oxaliplatin/Capecitabine. Targeted therapy drugs may be used towards stage IV adenocarcinoma tumors at the gastroesophageal junction. Trastuzumab is used against HER2-positive tumors in combination with Cisplatin/Capecitabine or Cisplatin/Fluorouracil [308]. Pembrolizumab improves the Oxaliplatin/S-1 therapy [88]. The more effective is Oxaliplatin/Fluorouracil/Leucovorin [86] with the addition of Docetaxel [34,95], or of antibodies Onartuzumab [86], Panitumumab [87], Rilotumumab [87].

#### 4.3.5. Gastric Cancer

Gastric cancer is the fifth most frequently diagnosed cancer and the third leading cause of cancer death, with rates two-fold higher in men than in women [305]. Different double regimens for the therapy of gastric cancer has been introduced in clinical practice:

(1) Cisplatin [36], or Oxaliplatin [89] with Capecitabine;
(2) Carboplatin [74], or Lobaplatin [166] with Paclitaxel;
(3) Oxaliplatin/Pemetrexed (PEMOX) [220];
(4) Satraplatin/Gemcitabine [174].

For the treatment of gastric cancer, the additional drug leads to the improvement of the efficacy of double schemes and the survival of patients. The most commonly applied triple schedules for gastric cancer are:

(1) Cisplatin/Capecitabine with Epirubicin [309], or Paclitaxel [37];
(2) Cisplatin/Leucovorin/Epirubicin [309];
(3) Cisplatin/S-1/Trastuzumab [38];
(4) Heptaplatin, Fluorouracil with Leucovorin [142], or Paclitaxel [310].

For an advanced gastric cancer, the reference combination is Cisplatin/Fluorouracil [220]. In a phase III study, it was proved that the addition of Doxetacel or Epirubicin [220], or Epirubicin/Leucovorin is more effective than the reference Cisplatin/Fluorouracil alone. The simultaneous use of Epirubicin increases the therapeutic effect of Cisplatin or Carboplatin with Capecitabine or Fluorouracil [220]. The survival efficacy of Oxaliplatin/Fluorouracil is enhanced from the application of Epirubicin [91], Leucovorin [94], Avelumab [96]. A better effect is obtained from the addition to Oxaliplatin/Capecitabine of Epirubicin [220], Docetaxel [90,91], Sintilimab [85], or Trastuzumab [92]. Promising results are achieved by the addition to Oxaliplatin/(S-1) [97] of Paclitaxel [311,312], Pembrolizumab [88], or Capecitabine/Nivolumab [93] and by the improvement the effect of Oxaliplatin/Fluorouracil by the addition of Leucovorin/Docetaxel [95].

#### 4.3.6. Colon Cancer

For stages II and III of colon cancer, the types of chemotherapy include the following regimens: CAPOX, SOX, FOLFOX:

(1) Oxaliplatin/Capecitabine (CAPOX, XELOX) [232,233];
(2) Oxaliplatin/Capecitabine/Bevacizumab [313];
(3) Oxaliplatin Capecitabine (CAPOX)/Erlotinib [313];
(4) Oxaliplatin/Fluorouracil/Leucovorin (Folinic acid (FOLFOX; FLOX} [232];
(5) Oxaliplatin/S-1 (SOX) [237].

The anticancer effect of CAPOX is optimized from simultaneous use of Erlotinib orBevacizumab [313]. For stage IV of colon cancer, better results than FOLFOX and FOLFIRI have been obtained from FOLFOXIRI, which is a scheme obtained from the addition of Irinotecan to FOLFOX or of Oxaliplatin to FOLFIRI [232]:

(1) Oxaliplatin/Fluorouracil/Leucovorin (Folinic acid) (FOLFOX; FLOX) [232];
(2) Oxaliplatin/Fluorouracil/Leucovorin/Irinotecan (FOLFOXIRI) [99];
(3) Fluorouracil/Leucovorin/Irinotecan (FOLFIRI).

#### 4.3.7. Colorectal Cancer

Colorectal cancer is one of the most common malignant neoplasms, showing, year by year, an increasing tendency in terms of both morbidity and deaths. In terms of incidence in the world and depending on the location, type of cancer, or gender, the disease is the third most commonly occurring cancer in men, the second most commonly occurring cancer in women, and is the second most common cause of death from cancer (9.2%) [314].

The effectivity of Oxaliplatin towards metastatic colorectal cancer is improved from the addition of Capecitabine [233], Capecitabine/Bevacizumab [233], Fluorouracil [234], or human–mouse monoclonal antibody Cetuximab (inhibitor of tumor growth and metastasis) [315]. As first-line therapy, CAPOX [233], SOX [316], (FOLFOX, FLOX), [316,317,318], or FOLFOXIRI [317] are applied. The anticancer effect obtained from FOLFOX is enhanced by simultianeous application with Irinotecan (FOLFOXIRI, FOLFIRINOX) [317], Atezolizumab [99], Bevacizumab [316,317,318], Tremelimumab or Panitumumab [100]. The activity of SOX [316] is improved by Bevacizumab [317]. Anticancer activity of Oxaliplatin/Fluorouracil/Leucovorin/Irinotecan (FOLFOXIRI) is improved by the addition of Bevacizumab [99,317] and Atezolizumab/Bevacizumab [99].

#### 4.3.8. Pancreatic Adenocarcinoma

Pancreatic cancer is one of the leading causes of cancer death worldwide and its global burden has more than doubled over the past 25 years, with the highest incidence in North America, Europe, and Australia [319]. Different chemotherapy schedules in treating of advanced or metastatic pancreatic cancer have been reported:

(1) Cisplatin/Capecitabine/Gemcitabine with: Docetaxel [39]; Nab-Paclitaxel [39]; Epirubicin [39];
(2) Oxaliplatin/Gemcitabine/Prednisolone [238];
(3) Oxaliplatin/Fluorouracil/Irinotecan/Leucovorin (FOLFIRINOX) [226];
(4) Oxaliplatin/Fluorouracil/Nab-Paclitaxel/Leucovorin/Bevacizumab [320].

The combination of Oxaliplatin with Fluorouracil results in higher response rates and longer survival thanwith either Oxaliplatin or Fluorouracil alone. The addition of Irinotecan to Oxaliplatin/Fluorouracil [235] improves outcomes in the treatment of patients and FOLFIRINOX has better short- and long-term efficacy [226].

#### 4.3.9. Ovarian and Endometrial Cancer

Ovarian cancer is the third most-frequent gynecological cancer and ranks fifth among cancer-related deaths in women. Malignant epithelial ovarian neoplasms are most ovarian tumors, the most common malignant ovarian neoplasm and are the eighth leading cause of cancer deaths worldwide, with 5-year survival rates below 45% [321].

For the improvement of the effect, for the first-line therapy of ovarian cancer, simultaneous application of Carboplatin [227], Oxaliplatin [268], or Nedaplatin [131] with Paclitaxel has been accepted as the standard treatment. Tubulin contributes to the mitosis, intracellular transport and cell shape. By binding to the specific sites on tubulin, Paclitaxel stabilizes microtubules, and as a result inhibits the normal microtubule reorganization and increases the effect of platinum drugs. The most common chemotherapy drug regimens against epithelial ovarian cancer are Cisplatin or Carboplatin with one of the following drugs: Cyclophosphamide [322], Gemcitabine, Docetaxel, Paclitaxel, Doxorubicin [227]. The most commondrug schemes for stromal cell ovarian cancer is Carboplatin/Paclitaxel [323]. For germ cell ovarian cancer, Carboplatin/Etoposideis administered [324]. For both stromal cell and germ cell ovarian cancer, Cisplatin/Etoposide/Bleomycin is applied [323,324].

In ovarian cancer, the advantage is the synergism between Cisplatin and nucleoside analog Gemcitabine [209], which increases platinum-DNA adduct formation and suppression of DNA synthesis by incorporation into DNA [209]. The oxazaphosphorine DNA-alkylating agent Cyclophosphamide is a prodrug, metabolized in the liver by CYP450 enzymes to active metabolites, binds to DNA to form interstrand and intrastrand crosslinks with lethal effect for cells. Through this mechanism, Cyclophosphamide improves the effectivity of the platinun drugs. The increased therapeutic effect has been reported with targeted or immunotherapy in addition to a platinum drug such as the following:

(1) Carboplatin or Oxaliplatin with Bortezomib [75];
(2) Carboplatin/Bevacizumab with: Docetaxel, Doxorubicin, or Gemcitabine [227];
(3) Carboplatin/Paclitaxel with Avelumab [325], Bevacizumab/Olaparib [227], Nintedanib [326], or Trebananib [327];
(4) Nedaplatin/Bevacizumab [132].

The most often adminisitered regimens for the treatment of endometrial cancer inlcude:

(1) Cisplatin with Paclitaxel [252], Doxorubicin [252], or Doxorubicin/Paclitaxel [328];
(2) Carboplatin with Doxorubicin [252], or Paclitaxel [252];
(3) Nedaplatin/Irinotecan [133].

#### 4.3.10. Breast and Cervical Cancer

Breast cancer is one of the leading causes cause of cancer in both sexes (11.6%), and occupies the fifth position in terms of mortality. Among females, this disease is the most commonly diagnosed cancer and the leading cause of cancer death, followed by colorectal and lung cancer (for incidence), and vice versa (for mortality). Cervical cancer ranks fourth for both incidence and mortality [274]. Both for breast and cervical cancer, the most often applied are different schemes between platinum agents and antimetabolite Gemcitabine [69,225,228], or with tuboline inhibitors: Paclitaxel [134,135,222,262] and Vinorebine [222,260,269] and the most common equal triple schedules are Carboplatin/Paclitaxel/Bevacizumab [329,330] and Carboplatin/Paclitaxel/Pembrolizumab [330,331] (Table 11).

For breast cancer-specific combinations, the following have been approved (Table 11):

(1) Capecitabine with Cisplatin [68], or Lobaplatin [161];
(2) Docetaxel with Cisplatin [259], Carboplatin [261], Nedaplatin [260], or Lobaplatin [162];
(3) Carboplatin/Gemcitabine with Iniparib [69] or Pembrolizumab [70];
(4) Carboplatin/Docetaxel/Trastuzumab/Pertuzumab [71].

For cervical cancer specific regimens, thereare (Table 11):

(1) Cisplatin with Ifosfamide, Mitomycin, Fluorouracil [222], S-1 [224], or Topotecan [209];
(2) Cisplatin or Carboplatin with Paclitaxel/Bevacizumab/Pembrolizumab [330].

#### 4.3.11. Prostate and Germ Cell Cancer

Prostate cancer is the most frequently diagnosed cancer among men in 105 countries of the world, and is the leading cause of cancer death among men in 46 countries. Germ cancer is common in the male population. Different schemes for treatment of prostate cancer have been designed (Table 12), which consist of a platinum drug and:

(1) antimetabolites: Fluorouracil [236], Gemcitabine [174,238], Pemetrexed [242];
(2) topoisomerase inhibitors: Etoposide [250], Epirubicin [250];
(3) taxanes: Cabazitaxel [265], Docetaxel [173,250], Paclitaxel [263];
(4) steroids: Prednisone [175,334];
(5) vascular endothelial growth factor receptors inhibitor Bevacizumab [172].

For the treatment of germ cell cancer, the following common schedules have been developed:

(1) Cisplatin/Ifosfamide with Gemcitabine [335], Etoposide [253], Paclitaxel [336], Vinblastine [253], or Methotrexate/Paclitaxel [337];
(2) Cisplatin/Bleomycin/Vincristine with Carboplatin [338];
(3) Cisplatin/Bleomycin/Etoposide [338,339,340] with Vincristine or Paclitaxel [341].

**Table 12:** Combinations of Cisplatin derivatives for therapy of prostate and germ cell cancers.

Prostate Cancer [250]	
(1) Cisplatin/Epirubicin [250] (2) Cisplatin/Etoposide [250] (3) Cisplatin/Prednisone [334] (4) Carboplatin/Epirubicin [250] (5) Carboplatin/Etoposide [250] (6) Carboplatin/Cabazitaxel [265] (7) Carboplatin/Docetaxel [250] (8) Carboplatin/Paclitaxel [263]	(9) Oxaliplatin/Fluorouracil [236] (10) Oxaliplatin/Gemcitabine [238] (11) Oxaliplatin/Gemcitabine/Prednisolone [238] (12) Oxaliplatin/Pemetrexed [242] (13) Satraplatin/Gemcitabine [174] (14) Satraplatin/Docetaxel [173] (15) Satraplatin/Prednisone [175] (16) Satraplatin/Bevacizumab [172]
**Germ Cell Cancers**	
(1) Csplatin/Epirubicin [253] (2) Cisplatin/Etoposide [251] (3) Cisplatin/Peplomycin/Methotrexate/Etoposide/Vincristine [253] (4) Cisplatin/Cyclophosphamide/Doxorubicin [339] (5) Cisplatin/Cyclophosphamide/Bleomycin/ Dactinomycin/Vinblastine [251] (6) Cisplatin/Ifosfamide/Gemcitabine (GIP) [335] (7) Cisplatin/Ifosfamide/Methotrexate/Paclitaxel (M-TIP) [337] (8) Cisplatin/Ifosfamide/Etoposide (VIP) [253] (9) Cisplatin/Ifosfamide/Paclitaxel [336] (10) Cisplatin/Ifosfamide/Vinblastine (VeIP) [253] (11) Cisplatin/Bleomycin/Vincristine (BOP) [338]	(12) Cisplatin/Bleomycin/Vincristine/Carboplatin [338] (13) Cisplatin/Bleomycin/Etoposide (BEP) [338,339,340] (14) Cisplatin/Bleomycin/Etoposide/Vincristine [253] (15) Cisplatin/Bleomycin/Etoposide/Paclitaxel [341] (16) Carboplatin/Etoposide [253] (17) Carboplatin/Ifosfamide/Etoposide/Paclitaxel) [253] (18) Carboplatin/Gemcitabine/Veliparib [342] (19) Oxaliplatin and gemcitabine [239] (20) Oxaliplatin/Gemcitabine/Paclitaxel [343] (21) Oxaliplatin/Bevacizumab [344] (22) Satraplatin/Gemcitabine [174]

#### 4.3.12. Bladder Cancer

The administered therapeutic regimens against germ cell cancer are:

(1) Cisplatin with Fluorouracil [221], Gemcitabine [221], Methotrexate [221], or Paclitaxel [221];
(2) Cisplatin/Fluorouracil/Mitomycin [222];
(3) Cisplatin/Gemcitabine with/Bevacizumab [222], or Pembrolizumab [345];
(4) Cisplatin/Methotrexate with Doxorubicin, Vinblastine, Doxorubicin/Vinblastine or Doxorubicin/Vincristine [222];
(5) Cisplatin or Carboplatin with Gemcitabine/Atezolizumab [222];
(6) Carboplatin/Paclitaxel [221];
(7) Satraplatin/Gemcitabine [174].

#### 4.3.13. Lymphoma, Carcinoma, Sarcoma, Blastoma, Mesothelioma, Melanoma

Other more common types of cancer include:

(1) Lymphomas: non-Hodgkin lymphoma (type: refractory diffuse large B-cell lymphoma); peripheral T-cell lymphoma (type: anaplastic large cell lymphoma);
(2) Carcinomas: conjunctival and oral squamous cell carcinomas; nasopharyngeal, salivary gland, neuroendocrine lung, head and neck, thymic, sarcomatoid, basaloid, mucoepidermoid, papillary, undifferentiated; hepatocellular, cervical. endometrial, urothelial, anal squamous cell, and penile carcinoma;
(3) Sarcomas: osteosarcoma, rhabdomyosarcoma;
(4) Multiple myeloma, mesothelioma;
(5) Blastomas: neuro-, retino-, and hepatoblastoma.

## 5. Lymphomas

Relapsed or refractory diffuse large B-cell lymphoma (DLBCL) is the most common and aggressive subtype of non-Hodgkin lymphoma, against which the following common treatment schemes have been introduced [346,347] (Table 13):

(1) Cisplatinor Oxaliplatin with Gemcitabine (GEMOX R);
(2) Cisplatin/Cytarabine with one of the following drugs: Fludarabin, Rituximab [348], Dexamethasone (DHAP) [348], Gemcitabine/Dexamethasone (GDP), Fludarabine/Mitoxantrone (MIFAP), or Etoposide/Methylprednisolone (ESHAP) [347];
(3) Cisplatin/Cytarabine/Dexamethasone (DHAP) with Decitabine [346], Mitoxantrone, or Rituximab (R-DHAP) [348];
(4) Carboplatin/Ifosfamide/Etoposide (ICE) [349] with Rituximab (R-ICE), or Dexamethasone (DICE).

The addition to all of these schemes of Rituximab, increases the anticancer effect. Decitabine is a specific DNA methyltransferase inhibitor that exerts antitumor effects by re-expressing tumor suppressor genes. The survival of patients after treatment with Decitabine to Cisplatin/Cytarabine/Dexamethasone (DHAP) is enhanced after the addition of Rituximab to DHAP [346]. Based on in vitro synergism, the combination of Cisplatin/Cytarabine (Ara-C) is the basis of many schedules for patients with aggressive non-Hodgkin lymphoma. It is suggested that the addition of Fludarabine as a potential biochemical modulator and may enhance the activity of Cisplatin/Ara-C [350]. Mitoxantrone increases the activity of DHAP and Cisplatin/Cytarabine/Fludarabine, promising combination is Carboplatin/Docetaxel/Trastuzumab/Pertuzumab (TCH).

The primary systemic type of anaplastic large cell lymphoma (ALCL) is a common subtype of the heterogeneous group of peripheral T-cell lymphomas (PTCL), which is characterized by large pleomorphic cells with strong expression of CD30. A regimens, consisting of the anti-CD30 drug Brentuximab vedotin, and classical lymphoma scheduleDHAP (Cisplatin/Cytarabine/Dexamethasone), show considerable cancer regression [351].

**Table 13:** Combinations of Cisplatin derivatives for therapy of lymphomas.

**Non-Hodgkin lymphoma** [352] (1) Cisplatin/Gemcitabine/Dexamethasone (GDP) (2) Cisplatin/Gemcitabine/Dexamethasone/Rituximab (R-GDP) (3) Cisplatin/Cytarabine/Fludarabine (4) Cisplatin/Cytarabine/Rituximab [348] (5) Cisplatin/Cytarabine/Dexamethasone (DHAP) [348] (6) Cisplatin/Cytarabine/Dexamethasone (DHAP) [348] with Rituximab (R-DHAP) [348] (7) Cisplatin/Cytarabine/Dexamethasone/Decitabine [346] (8) Cisplatin/Cytarabine/Dexamethasone/Mitoxantrone [352] (9) Cisplatin/Cytarabine/Etoposide/Methylprednisolone (ESHAP) (10) Cisplatin/Cytarabine/Etoposide/Methylprednisolone/Rituximab (R-ESHAP) (11) Cisplatin/Cytarabine/Fludarabine/Mitoxantrone (MIFAP) (12) Carboplatin/Ifosfamide/Etoposide (ICE) [349] (13) Carboplatin/Ifosfamide/Etoposide/Rituximab (R-ICE) (14) Carboplatin/Ifosfamide/Etoposide/Dexamethasone (DICE) (15) Carboplatin/Docetaxel/Trastuzumab/Pertuzumab (16) Oxaliplatin/Gemcitabine (GEMOX R) (17) Oxaliplatin/Gemcitabine/Rituximab
**Peripheral T-cell lymphoma—type: anaplastic large cell lymphoma** (1) Cisplatin/Gemcitabine/Dexamethasone [353] (2) Cisplatin/Gemcitabine/Dexamethasone/Pegaspargase [354] (3) Cisplatin/Cytarabine/Dexamethasone) (DHAP) [353] (4) Cisplatin/Cytarabine/Dexamethasone/Brentuximab vedotin [351]
**Lymphoepithelioma** (1) Nedaplatin/Paclitaxel/Nivolumab [115]

## 6. Carcinomas 

In a phase III study of advanced head and neck carcinoma, the comparison of Cisplatin/Fluorouracil with Cisplatin/Fluorouracil/Paclitaxel induction chemotherapy shows that the addition of Paclitaxel leads to more beneficial results than the double standard schedule, and inclusion of radiation improves the effect of the triple combination and is the more effective [217]. Improved therapeutic effect is obtained with addition to Cisplatin/Fluorouracil of Docetaxel [355], Paclitaxel [217], Cetuximab [21], Cetuximab/Docetaxel [21], or in addition to Carboplatin/Fluorouracil [56] of Docetaxel [355] or Cetuximab [21]. Nedaplatin regimens with Capecitabine [243], Fluorouracil [123], Gemcitabine [122], or Paclitaxel/S-1 [124] are the most often double schemes for nasopharyngeal carcinoma (Table 14).

Thymic carcinoma is a rare neoplasm that often disseminates or metastasizes. Various histopathological subtypes have been described, which include epidermoid keratinizing and non-keratinizing carcinoma, clear cell carcinoma, lymphoepithelioma-like carcinoma, sarcomatoid, basaloid, mucoepidermoid, and papillary carcinoma. An effective treatment for advanced thymic carcinoma consists of Carboplatin/Paclitaxel [356] or addition to Cisplatin/Doxorubicin of Cyclophosphamide [357], or Etoposide/Vincristine [358].

Liver cancer is the sixth most commonly diagnosed and the fourth leading cause of cancer death worldwide with rates of both incidence and mortality two to three times higher among men in most world regions. Liver cancer ranks fifth in terms of global cases and second in terms of deaths for males.

For cholangiocarcinoma treatment, the most important schedules used are:

(1) Cisplatin/Gemcitabine [40,359] with S-1 [359] or Nab-Paclitaxel [360];
(2) Cisplatin/Fluorouracil/Epirubicin [361];
(3) GEMOX [362,363,364] with Bevacizumab [365] or Erlotinib [364];
(4) FOLFOX [366].

The therapeutic effect of Oxaliplatin/Capecitabine against hepatocellular carcinoma is enhanced by the addition of Bevacizumab [367]. For the obtaining of improved results are applied combinations: FOLFOX) [368] alone or with Sorafenib [369] or GEMOX alone [370] or with Sorafenib [371].

As the standard treatment for advanced urothelial carcinoma currently the preferred first-line chemotherapy is Cisplatin/Gemcitabine, due this regimens leads to long-term survival results and is less toxic than the other applied MVAC regimen Cisplatin/Methotrexate/Doxorubicin/Vinblastine [372]. New current data suggests that, for effective and safe control of urothelial carcinoma, thecompensatory scheme of Cisplatin/Gemcitabine/Romidepsin [373].

Towards penile carcinoma very often schedules applied are:

(1) Cisplatin with Irinotecan [248] or Cemiplimab [44];
(2) Cisplatin/Fluorouracil with Doxetacel or Paxlitacel [374].

**Table 14:** Combinations of Cisplatin derivatives for therapy of carcinomas.

**Head and neck squamous cell carcinoma** (1) Cisplatin/Fluorouracil [56,217,355] (2) Cisplatin/Fluorouracil/Docetaxel [355] (3) Cisplatin/Fluorouracil/Docetaxel/Cetuximab [21] (4) Cisplatin/Fluorouracil/Paclitaxel [217] (5) Cisplatin/Fluorouracil/Cetuximab [21] (6) Carboplatin/Fluorouracil [56] (7) Carboplatin/Fluorouracil/Docetaxel [355] (8) Carboplatin/Fluorouracil/Cetuximab [21] (9) Carboplatin/Paclitaxel/Cetuximab [21] (10) Nedaplatin/Fluorouracil [209] (11) Nedaplatin/S-1 [108]	**Cholangiocarcinoma (biliary-tract cancer)** (1) Cisplatin/Fluorouracil/Epirubicin [361] (2) Cisplatin/Capecitabine [40] (3) Cisplatin/Gemcitabine [40,359] (4) Cisplatin/Gemcitabine/S-1 (GCS) [359] (5) Cisplatin/Gemcitabine/Nab-Paclitaxel [360] (6) Oxaliplatin/Capecitabine [362] (7) Oxaliplatin/Fluorouracil/Leucovorin (FOLFOX) [366] (8) Oxaliplatin/Gemcitabine [362,363,364] (9) Oxaliplatin/Gemcitabine/Bevacizumab [365] (10) Oxaliplatin/Gemcitabine/Erlotinib [364]
**tNasopharyngeal carcinoma** (1) Cisplatin/Bleomycin/Methotrexate (BMP) (2) Cisplatin/Fluorouracil [219] (3) Cisplatin/Fluorouracil/Docetaxel [219] (4) Nedaplatin Capecitabine [243] (5) Nedaplatin/Fluorouracil [123] (6) Nedaplatin/Gemcitabine [122] (7) Nedaplatin/Paclitaxel/S-1 [124] (8) Lobaplatin/Fluorouracil [156,157]	**Hepatocellularcarcinoma** (1) Oxaliplatin/Capecitabine/Bevacizumab [367] (2) Oxaliplatin/Fluorouracil/Leucovorin (FOLFOX) [368] (3) Oxaliplatin/Fluorouracil/Leucovorin/Sorafenib [369] (4) Oxaliplatin/S-1 [102] (5) Oxaliplatin/Gemcitabine (GEMOX) [370] (6) Oxaliplatin/Gemcitabine/Sorafenib [371] (7) Satraplatin/Gemcitabine [174]
**Thymic carcinoma** (1) Cisplatin/Cyclophosphamide/Doxorubicin [357] (2) Cisplatin/Etoposide/Doxorubicin/Vincristine [358] (3) Carboplatin/Paclitaxel [356] **Conjunctival squamous cell carcinoma** (1) Cisplatin/Fluorouracil [218] **Oral squamous cellcarcinoma** (1) Nedaplatin/Docetaxel [209] **Salivary gland carcinoma** (1) Carboplatin/Gemcitabine [54] **Neuroendocrine lung carcinoma** (1) Nedaplatin/Irinotecan [114] **Cutaneous cell carcinoma** (1) Cisplatin/Bleomycin (2) Cisplatin/Doxorubicin (3) Cisplatin/Paclitaxel [375]	**Urothelial carcinoma** (1) Cisplatin/Gemcitabine [229] (2) Cisplatin/Gemcitabine/Everolimus [41] (3) Cisplatin/Gemcitabine/Romidepsin [373] (4) Cisplatin/Methotrexate/Doxorubicin/Vinblastine [372] (5) Carboplatin/Paclitaxel [70] (6) Carboplatin/Paclitaxel/Trastuzumab [76] (7) Nedaplatin/Gemcitabine [129] (8) Nedaplatin/Paclitaxel/Ifosfamide [130] **Penilecarcinoma** (1) Cisplatin/Ifosfamide/Paclitaxel [374] (2) Cisplatin/Fluorouracil/Doxetael [374] (3) Cisplatin/Fluorouracil/Paxlitacel [374] (4) Cisplatin/Irinotecan [248] (5) Cisplatin/Cemiplimab [44] **Anal squamous cell carcinoma** (1) Cisplatin/Capecitabine/radiation therapy [42] (2) Cisplatin/Fluorouracil/Docetaxel [43] (3) Cisplatin/Fluorouracil/Docetaxel/Atezolizumab [43]

## 7. Sarcoma

The development and prognosis of osteosarcoma are pathological processes, involving multiple genes and factors [376]. For the treatment of osteosarcoma the standard multidrug chemotherapy consists of Cisplatin/Methotrexate/Doxorubicin [377,378], theactivity of which is enhanced in addition of Ifosfamide [379] or Etoposide [378]. Another important regimen is Cisplatin/Ifosfamide/Epirubicin [380].

## 8. Blastoma

Therapy schemes for pediatric solid tumors have been developed:

(1) Neuroblastoma: Cisplatin/Etoposide/Cyclophosphamide/Doxorubicin/Vincristine;
(2) Retinoblastoma: Carboplatin/Etoposide/Vincristine (VEC);
(3) Hepatoblastoma: Cisplatin/Fluorouracil/Vincristine with or without Doxorubicin [381].

## 9. Mesothelioma

For the chemotherapy of patients with advanced malignant pleural mesothelioma, Cisplatin/Raltitrexed [382] or Cisplatin/Pemetrexed [383] with Bevacizumab [384] or Nintedanib [385] are often administered.

## 10. Melanoma

For melanoma therapy, the following schedules are administered:

(1) Cisplatin/Dacarbazine [386] with Paclitaxel [387], Vindesine [388], or Interferon alpha-2b/Interleukin-2 [386];
(2) Cisplatin/Temozolomide/Interferon alpha-2b [389];
(3) Carboplatin/Paclitaxel [390] with Sorafenib [390] or Bevacizumab [391];
(4) Carboplatin/Nab-Paclitaxel/Bevacizumab [392].

In different cancers, the optimized effect of mono– and double combinations with radiotherapy have been observed:

(1) Cisplatin/Capecitabine—anal squamous cell carcinoma [42];
(2) Cisplatin/Fluorouracil/Docetaxel—head and neck cancer [217];
(3) Cisplatin/Fluorouracil/Paclitaxel—head and neck cancer [217];
(4) Carboplatin/Gemcitabine; Carboplatin/Vinorelbine—NSCLC [206];
(5) Carboplatin/Irinotecan;
(6) Carboplatin/Fluorouracil/Paclitaxel—esophageal cancer [209];
(7) Nedaplatin—nasopharyngeal carcinoma [125].

Melanoma represents the most aggressive skin cancer [393] and is responsible for most cutaneous malignancy-related deaths. An intracellular enzyme paraoxonase-2 posseses a protective role toward reactive oxygen formation within mitochondrial respiratory chain and could be a novel biomarker for the targeted anticancer therapy against melanoma [394] and bladder cancer [395].

Combinations with encapsulated Cisplatin derivatives are very promising. Nanocarriers provide improved delivery of platinum anticancer drugs, reduced drug influx, improvement of intracellular penetration, increased concentration of platinum drugs in cancer cells. The development of new nanotechnological drug systems for the targeted delivery of cytotoxic drugs and selective accumulation in tumor tissue leads to enhanced cytotoxicity, enhancement of therapeutic efficacy, overcomimg the drug resistance, and reduction of the toxicity of the anticancer agents [396]. Nanocarriers are:

I. Liposomally encapsulated Cisplatin alone and combined with Gemcitabine [397] or Paclitaxel [396];
II. Liposomally encapsulated Oxaliplatin [398].
  Combinations of Lipoplatin have been investigated with the following drugs:
  (1) Gemcitabine in metastatic pancreatic cancer, bladder, neck, head, non-small-cell lung cancer [397], malignant pleural mesothelioma;
  (2) Fluorouracil in phase III in squamous cell carcinoma of the head and neck and in advanced gastric cancer [399] with 80% efficiency;
  (3) Docetaxel or Navelbine in metastatic breast cancer;
  (4) Paclitaxel in non-small-cell lung cancer—the response rate is similar, but nephrotoxicity, neurotoxicity and myelotoxicity are significantly lower. The main dose-limiting toxicity is myelosuppression (neutropenia and thrombocytopenia);
  (5) Lipoplatin/Gemcitabine; Lipoplatin/Docetaxel for NSCLC;
  (6) Lipoplatin/Docetaxel against metastatic breast cancer;
  (7) Lipoplatin/radiation towards head and neck cancers.

SPI-77 is a sterically stabilized liposome Cisplatin, used in osteosarcoma of the head and neck, and with Vinorelbine has been applied against non-small-cell lung cancer, with minimal granulocytopenia and thrombocytopenia, oto-, neuro- and nephrotoxicity. The main toxicity is neutropenia [400].

Combination chemotherapy involves simultaneously treatment with many different drugs, with different mechanisms and side-effects. This fact provides the biggest advantages of combination regiments such as minimizing the resistance development to any one agent and the application at lower doses, which resulted in a reduction of toxicity.

## 11. Conclusions

The problems with anticancer therapy are resistance and toxicity. The trend for the improvement of the anticancer effect is a synergistic combination of different drug molecular mechanisms. In various treatment regimens, applied platinum agents with radiation or in combination with the following drugs are applied:

(1) Anticancer agents: Fluorouracil, Gemcitabine, Fludarabine, Pemetrexed, Ifosfamide, Irinotecan, Topotecan, Etoposide, Amrubicin, Doxorubicin, Epirubicin, Vinorelbine, Docetaxel, Paclitaxel, and Nab-Paclitaxel;
(2) Modulators of resistant mechanisms;
(3) Signaling protein inhibitors: Erlotinib; Bortezomib; and Everolimus;
(4) Immunotherapeutic drugs: Atezolizumab, Avelumab, Bevacizumab, Cemiplimab, Cetuximab, Durvalumab, Erlotinib, Imatinib, Necitumumab, Nimotuzumab, Nivolumab, Onartuzumab, Panitumumab, Pembrolizumab, Rilotumumab, Trastuzumab, Tremelimumab, and Sintilimab.

The advantages of the reported drug regimens and the rationality of combination therapy at the molecular level are:

(1) Achieving the maximum possible removal of tumor cells within the permissible toxicity;
(2) Affecting the cells located in different phases of the cell cycle;
(3) Realization of prevention or delay of the development of resistant cell branches;
(4) Slowing the process of adaptation of tumor cells and delay of cell mutations, due to using more drugs that have different molecular targets;
(5) Reduction of dose of combined components with one and the same dose-limiting effects;
(6) Schemes with components with different toxicity to normal tissues, allowing the application of optimal doses of each drug in the absence of superimposition of toxic effects on an organ or cell system.

An important approach for overcoming the drug resistance and for the reduction of toxicityis the combibation therapy of liposomally encapsulated Cisplatin and Oxaliplatinwith other anticancer agents, which provide improved targeted delivery, improved intracellular penetration, selective accumulation in tumor tissue, and enhanced therapeutic efficacy.

## Figures and Tables

**Table 1:** Cisplatin synergistic combinations.

Cisplatin Synergistic Combinations	Disease
(1) Cisplatin/Fluorouracil/Docetaxel/Cetuximab [21]	head and neck squamous cell carcinoma
(2) Cisplatin/Gemcitabine [22] (3) Cisplatin/Gemcitabine/Necitumumab [23]	squamous non-small-cell lung cancer
(4) Cisplatin/Vinorelbine [24]	non-squamous non-small-cell lung cancer
(5) Cisplatin/Etoposide/Irinotecan [25] (6) Cisplatin/Etoposide/Durvalumab [26] (7) Cisplatin/Vinorelbine [27]	small-cell lung cancer
(8) Cisplatin/Capecitabine [28] (9) Cisplatin/Cetuximab [29]	breast cancer
(10) Cisplatin/Docetaxel [30] (11) Cisplatin/Paclitaxel [31] (12) Cisplatin/Tegafur-Gimeracil-Oteracil potassium (S-1) [32]	esophageal squamous cell carcinoma
(13) Cisplatin/Capecitabine/Docetaxel [33] (14) Cisplatin/Capecitabine/Epirubicin [34] (15) Cisplatin/Capecitabine/Epirubicin/Rilotumumab [35] (16) Cisplatin/Fluorouracil/Epirubicin [34]	gastroesophageal adenocarcinoma
(17) Cisplatin/Capecitabine [36] (18) Cisplatin/Capecitabine/Paclitaxel [37] (19) Cisplatin/Tegafur-Gimeracil-Oteracil potassium (S-1) [36] (20) Cisplatin/Tegafur-Gimeracil-Oteracil potassium (S-1)/Trastuzumab [38]	gastric cancer
(21) Cisplatin/Capecitabine/Gemcitabine/Docetaxel [39] (22) Cisplatin/Capecitabine/Gemcitabine/Epirubicin [39] (23) Cisplatin/Capecitabine/Gemcitabine/Nab-Paclitaxel [39]	pancreatic adenocarcinoma
(24) Cisplatin/Capecitabine [40] (25) Cisplatin/Gemcitabine [40]	biliary cancer
(26) Cisplatin/Gemcitabine/Everolimus [41]	urothelial carcinoma
(27) Cisplatin/Capecitabine/radiation therapy [42] (28) Cisplatin/Fluorouracil/Docetaxel [43] (29) Cisplatin/Fluorouracil/Docetaxel/Atezolizumab [43]	anal squamous cell carcinoma
(30) Cisplatin/Cemiplimab [44]	metastatic penile cancer

**Table 2:** Carboplatin synergistic combinations.

Carboplatin Synergistic Combinations	Disease
(1) Carboplatin/Gemcitabine [54]	salivary gland cancer
(2) Carboplatin/Fluorouracil [56] (3) Carboplatin/Paclitaxel/Cetuximab [21]	head and neck squamous cell carcinoma
(4) Carboplatin/Gemcitabine [57] (5) Carboplatin/Gemcitabine/Avelumab [58] (6) Carboplatin/Paclitaxel [59] (7) Carboplatin/Nab-Paclitaxel/Atezolizumab [60] (8) Carboplatin/Paclitaxel/Bevacizumab [61] (9) Carboplatin/Paclitaxel/Nivolumab [62] (10) Carboplatin/Pemetrexed [61] (11) Carboplatin/Pemetrexed/Avelumab [58] (12) Carboplatin/Tegafur-Gimeracil-Oteracil potassium (S-1) [63] (13) Carboplatin/Vinorelbine [64]	non-small-cell lung cancer
(14) Carboplatin/Amrubicin [65] (15) Carboplatin/Etoposide [66] (16) Carboplatin/Etoposide/Atezolizumab [26,67] (17) Carboplatin/Etoposide/Durvalumab [26,66] (18) Carboplatin/Etoposide/Durvalumab/Tremelimumab [66] (19) Carboplatin/Etoposide/Obatoclax mesylate [68] (20) Carboplatin/Irinotecan [65]	small-cell lung cancer
(21) Carboplatin/Gemcitabine [69] (22) Carboplatin/Gemcitabine/Iniparib [69] (23) Carboplatin/Gemcitabine/Pembrolizumab [70] (24) Carboplatin/Docetaxel/Trastuzumab [71]	breast cancer
(25) Carboplatin/Paclitaxel [72]	esophageal squamous cell carcinoma
(26) Carboplatin/Docetaxel/Capecitabine [73]	gastroesophageal adenocarcinoma
(27) Carboplatin/Paclitaxel [74]	gastric cancer
(28) Carboplatin/Bortezomib [75]	ovarian cancer
(29) Carboplatin/Paclitaxel [70] (30) Carboplatin/Paclitaxel/Trastuzumab [76]	urotelial carcinoma

**Table 3:** Oxaliplatin synergistic combinations.

Oxaliplatin Synergistic Combinations	Disease
(1) Oxaliplatin/Cytarabine/Fludarabine [82]	leukemia
(2) Oxaliplatin/Cytarabin/Docetaxel [83]	non-small-cell lung cancer
(3) Oxaliplatin/Capecitabine [72] (4) Oxaliplatin/Fluorouracil/Leucovorin [84]	esophageal adenocarcinoma
(5) Oxaliplatin/Capecitabine/Epirubicin [73] (6) Oxaliplatin/Capecitabine/Sintilimab [85] (7) Oxaliplatin/Fluorouracil/Leucovorin [86] (8) Oxaliplatin/Fluorouracil/Leucovorin/Docetaxel [34] (9) Oxaliplatin/Fluorouracil/Leucovorin/Onartuzumab [86] (10) Oxaliplatin/Fluorouracil/Leucovorin/Panitumumab [87] (11) Oxaliplatin/Fluorouracil/Leucovorin/Rilotumumab [87] (12) Oxaliplatin/Tegafur-Gimeracil-Oteracil potassium (S-1)/Pembrolizumab [88]	gastroesophageal adenocarcinoma
(13) Oxaliplatin/Capecitabine [89] (14) Oxaliplatin/Capecitabine/Docetaxel [90,91] (15) Oxaliplatin/Capecitabine/Sintilimab [85] (16) Oxaliplatin/Capecitabine/Trastuzumab [92] (17) Oxaliplatin/Capecitabine/Tegafur-Gimeracil-Oteracil potassium (S-1)/Nivolumab [93] (18) Oxaliplatin/Fluorouracil/Epirubicin [91] (19) Oxaliplatin/Fluorouracil/Leucovorin [94] (20) Oxaliplatin/Fluorouracil/Leucovorin/Docetaxel [95] (21) Oxaliplatin/Fluorouracil/Avelumab [96] (22) Oxaliplatin/Tegafur-Gimeracil-Oteracil potassium (S-1) [97] (23) Oxaliplatin/Tegafur-Gimeracil-Oteracil potassium (S-1)/Pembrolizumab [88]	gastric cancer
(24) Oxaliplatin/Fluorouracil/Irinotecan/Leucovorin [98] (25) Oxaliplatin/Fluorouracil/Irinotecan/Bevacizumab [99] (26) Oxaliplatin/Fluorouracil/Leucovorin/Atezolizumab [99] (27) Oxaliplatin/Fluorouracil/Leucovorin/Bevacizumab [99] (28) Oxaliplatin/Fluorouracil/Leucovorin/Durvalumab [100] (29) Oxaliplatin/Fluorouracil/Leucovorin/Tremelimumab [100] (30) Oxaliplatin/Trifluridine/Tipiracil Limagne [101]	colorectal cancer
(31) Oxaliplatin/Tegafur-Gimeracil-Oteracil potassium (S-1) [102]	hepatocellular carcinoma
(32) Oxaliplatin/Bortezomib [75]	ovarian cancer

**Table 4:** Nedaplatin synergistic combinations.

Nedaplatin Synergistic Combinations	Disease
(1) Nedaplatin/Tegafur-Gimeracil-Oteracil potassium (S-1) [108]	head and neck squamous cell carcinoma
(2) Nedaplatin/Docetaxel [109] (3) Nedaplatin/Docetaxel/Paclitaxel [83] (4) Nedaplatin/Gemcitabine [110] (5) Nedaplatin/Irinotecan [111] (6) Nedaplatin/Nab-Paclitaxel [112] (7) Nedaplatin/Paclitaxel/Sintilimab [113]	non-small-cell lung cancer
(8) Nedaplatin/Irinotecan [114]	neuroendocrine lung carcinoma
(9) Nedaplatin/Paclitaxel/Nivolumab [115]	lymphoepithelioma
(10) Nedaplatin/Docetaxel [116] (11) Nedaplatin/Docetaxel/Fluorouracil [117] (12) Nedaplatin/Docetaxel/Tegafur-Gimeracil-Oteracil potassium (S-1) [118] (13) Nedaplatin/Fluorouracil [119] (14) Nedaplatin/Paclitaxel [120] (15) Nedaplatin/Paclitaxel/Nimotuzumab [121] (16) Nedaplatin/Gemcitabine/Nimotuzumab [121] (17) Nedaplatin/Fluorouracil/Nimotuzumab [121]	esophageal squamous cell carcinoma
(18) Nedaplatin/Gemcitabine [122] (19) Nedaplatin/Fluorouracil [123] (20) Nedaplatin/Paclitaxel/Tegafur-Gimeracil-Oteracil potassium (S-1) [124] (21) Nedaplatin/radiotherapy [125]	nasopharyngeal carcinoma
(22) Nedaplatin/Docetaxel [126] (23) Nedaplatin/Gemcitabine [127] (24) Nedaplatin/Irinotecan [128]	squamous lung cell carcinoma
(25) Nedaplatin/Gemcitabine [129] (26) Nedaplatin/Paclitaxel/Ifosfamide [130]	urothelial cancer
(27) Nedaplatin/Paclitaxel [131] (28) Nedaplatin/Bevacizumab [132]	ovarian cancer
(29) Nedaplatin/Irinotecan [133]	endometrial carcinoma
(30) Nedaplatin/Paclitaxel [134,135]	cervical cancer

**Table 5:** Lobaplatin synergistic combinations.

Lobaplatin Synergistic Combinations	Disease
(1) Lobaplatin/Fluorouracil [156,157]	nasopharyngeal carcinoma
(2) Lobaplatin/Etoposide [158] (3) Lobaplatin/Irinotecan [159]	small-cell lung cancer
(4) Lobaplatin/Pemetrexed/Bevacizumab/Temozolomide [160]	lung adenocarcinoma
(5) Lobaplatin/Capecitabine [161] (6) Lobaplatin/Docetaxel [162]	breast cancer
(7) Lobaplatin/Fluorouracil [163] (8) Lobaplatin/Fluorouracil/Leucovorin [164] (9) Lobaplatin/Paclitaxel [165]	esophageal carcinoma
(10) Lobaplatin/Paclitaxel [166]	gastric cancer

**Table 6:** Dose-limiting effects of Cisplatin its derivatives.

Toxicity	Drugs
Neurotoxicity	Cisplatin [51], Carboplatin [51], Oxaliplatin [51]
Nephrotoxicity	Cisplatin [178], Heptaplatin [179]
Ototoxicity	Cisplatin [180]
Myelosuppression (thrombocytopenia)	Carboplatin [51], Oxaliplatin [104], Nedaplatin [105], Lobaplatin [152], Satraplatin [174]
Myelosuppression (neutropenia)	Carboplatin [51], Satraplatin [174]
Gastrointestinal toxicity	Oxaliplatin [104]

**Table 7:** Mechanisms of action of anticancer drugs, applied in combination therapy with platinum derivatives.

Drugs	Mechanism	Target
Cyclophosphamide, Ifosfamide, Mitomycin C	DNA alkylators	DNA breaks
Bleomycin, Dactinomycin, Peplomycin	DNA cross-link breaks	
Capecitabine, Fluorouracil, S-1	thymidilate synthase inhibition	DNA-related proteins
Gemcitabine	ribonucleotide reductase inhibition	
Pemetrexed	dihydrofolic reductase, thymidilate synthase, formyltransferase ribonucleotide inhibition	
Cytarabine, Fludarabine	DNA polymerase and ribonucleotide reductase inhibition	
Decitabine	DNA demethylation	
Methotrexate	dihydrofolic reductase inhibition	
Camptotecins: Belotecan, Irinotecan, Topotecan	topoisomerase I inhibition	
Etoposide, Amrubicin, Doxorubicin, Epirubicin	topoisomerase II inhibition	
Dexamethasone, Prednisolone, Prednisone	transcriptional interaction with specific genes	Modification of genes
Trastuzumab	HER 2 inhibition	Tumor cells membrane receptors
Cetuximab	epidermalgrowth factor receptors (EGFR) (Her-1) inhibition	
Bevacizumab	vascular endothelial growth factor receptors (VEGFR) inhibition	
Imatinib	tyrosine kinase inhibition	Tumor cells intracellular pathways
Cabazitaxel, Docetaxel, Paclitaxel, Nab-Paclitaxel	microtubule stabilization	
Viblastine, Vincristine, Vinorelbine	inhibition of microtubule polymerization	

**Table 8:** Combinations of Cisplatin derivatives with antimetabolites and with S-1.

Drugs	Cisplatin and Derivatives
Cisplatin	
Capecitabine	breast [28], gastric [36], and biliary cancer [40], esophageal carcinoma [216]
Fluorouracil	head and neck cancer [56,217], esophageal [216], conjunctival [218], and nasopharyngeal [219] squamous cell carcinoma [218], nasopharyngeal [220], bladder [221], and cervical cancer [222]
S-1	pancreatic [223], and cervical cancer [224]
Gemcitabine	breast [225], pancreatic [226], bladder [221], biliary [40], ovarian [227], cervical [228], and non-small-cell lung cancer [22], urothelial carcinoma [229]
Pemetrexed	non-squamous non-small-cell lung cancer [221,230]
Methotrexate	bladder cancer [221]
**Carboplatin**	
Fluorouracil	non-small-cell lung cancer [209], esophageal [216], and head and neck squamous cell carcinoma [56]
Gemcitabine	breast [69], ovarian [231], salivary gland [54], and non-small-cell lung cancer [57], urothelial carcinoma [229]
Pemetrexed	non-small-cell lung cancer [61]
**Oxaliplatin**	
Capecitabine	esophageal adenocarcinoma [72,216], gastric [89], colon [232], and colorectal cancer [233]
Fluorouracil	esophageal [216], colorectal [234], pancreatic [235], and prostate cancer [236]
S-1	colon cancer [237], hepatocellular carcinoma [102]
Gemcitabine	pancreatic [226], prostate [238], and germ cell cancer [239]
Pemetrexed	head and neck [240], gastric [220], colorectal [241], and prostate cancer [242]
**Nedaplatin**	
Capecitabine	nasopharyngeal carcinoma [243]
Fluorouracil	head and neck cancer [209], nasopharyngeal [123], and esophageal squamous cell carcinoma [119]
S-1	head and neck squamous cell carcinoma [108]
Gemcitabine	non-small-cell lung cancer [110], nasopharyngeal [122], urothelial [129], and lung squamous cell carcinoma [127]
**Lobaplatin**	
Capecitabine	breast cancer [161]
Fluorouracil	nasopharyngeal [156,157], and esophageal carcinoma [163]
**Satraplatin**	
Gemcitabine	prostate, gastric, pancreatic, bladder cancer, hepatocellular and papillary renal carcinoma [174]

**Table 11:** Combinations of Cisplatin derivatiives for therapy of breast and cervical cancer.

Breast Cancer	Cervical Carncer [222]
(1) Cisplatin/Capecitabine [28]	(1) Cisplatin/Ifosfamide
(2) Cisplatin/Gemcitabine [225]	(2) Cisplatin/Mitomycin
(3) Cisplatin/Docetaxel [259]	
(4) Cisplatin/Vinorebine [260]	(3) Cisplatin/Fluorouracil
(5) Cisplatin/Cetuximab [29]	(4) Cisplatin/S-1 [224]
(6) Cisplatin/Bevacizumab [332]	
(7) Cisplatin/Fluorouracil/Vinorelbine (VIFUP)	(5) Cisplatin/Gemcitabine [228]
(8) Carboplatin/Gemcitabine [69]	(6) Cisplatin/Topotecan [209]
(9) Carboplatin/Docetaxel [261]	
(10) Carboplatin/Paclitaxel [262]	(7) Cisplatin/Paclitaxel
(11) Carboplatin/Nab-Paclitaxel [264]	(8) Cisplatin/Vinorelbine
(12) Carboplatin/Vinorebine [260]	
(13) Carboplatin/Veliparib [333]	(9) Cisplatin/Paclitaxel/
(14) Carboplatin/Gemcitabine/Iniparib [69]	Bevacizumab/Pembrolizumab [330]
(15) Carboplatin/Gemcitabine/Pembrolizumab [70]	
(16) Carboplatin/Docetaxel/Trastuzumab [71]	(10) Carboplatin/Paclitaxel/
(17) Carboplatin/Docetaxel/Trastuzumab/Pertuzumab [71]	Bevacizumab/Pembrolizumab [330]
(18) Carboplatin/Paclitaxel/Bevacizumab [329]	
(19) Carboplatin/Paclitaxel/Pembrolizumab [331]	(11) Carboplatin/Paclitaxel/
(20) Oxaliplatin/Vinorebine [269]	Bevacizumab [330]
(21) Nedaplatin/Docetaxel [260]	
(22) Nedaplatin/Vinorebin [260]	(12) Carboplatin/Paclitaxel/
(23) Lobaplatin/Capecitabine [161]	Pembrolizumab [330]
(24) Lobaplatin/Docetaxel [162]	
(25) Lobaplatin/Vinorebine [260]	(13) Nedaplatin/Paclitaxel [134,135]
